# Aging‐Driven Immunosuppression: The Role of Tregs in the Ovarian Tumor Microenvironment

**DOI:** 10.1111/acel.70510

**Published:** 2026-04-24

**Authors:** Mary P. Udumula, Anjaly KM, Faraz Rashid, Mohammad Nematullah, Harshit Singh, Tanya Bhardwaj, Miriana Hijaz, Shailendra Giri, Eduardo N. Chini, Heather M. Gibson, Ramandeep Rattan

**Affiliations:** ^1^ Division of Gynecologic Oncology Henry Ford Hospital Detroit Michigan USA; ^2^ Henry Ford Cancer Institute Detroit Michigan USA; ^3^ Department of Obstetrics, Gynecology & Reproductive Biology Michigan State University Lansing Michigan USA; ^4^ Department of Neurology Henry Ford Hospital Detroit Michigan USA; ^5^ Department of Biology University of Michigan Ann Arbor Michigan USA; ^6^ Department of Cancer Biology Mayo Clinic Jacksonville Florida USA; ^7^ Department of Oncology Wayne State School of Medicine Detroit Michigan USA

**Keywords:** aging, immunosuppression, Tregs

## Abstract

Epithelial ovarian cancer (EOC) incidence and mortality increase with age, driven in part by chronic inflammation, diminished T cell output, and heightened regulatory T cell (Treg) mediated immunosuppression. In aged EOC‐bearing mice, we observed reduced survival, accompanied by impaired CD4^+^ and CD8^+^ T cell responses and a marked expansion of FOXP3^+^ Tregs exhibiting elevated IL‐10 and TGFβ expression. Metabolic profiling revealed enhanced oxidative phosphorylation in Tregs from aged mice, along with a fivefold increase in intracellular succinate levels. This accumulation of succinate within the aged tumor microenvironment was found to potentiate Treg suppressive function. Notably, pharmacologic inhibition of α‐ketoglutarate dehydrogenase reversed this effect, restoring effector T cell activity. These findings highlight succinate‐driven metabolic reprogramming as a central mechanism of age‐related Treg dysfunction in EOC and suggest that targeting succinate metabolism may offer a promising strategy to rejuvenate antitumor immunity in elderly patients.

## Graphical abstract

In the aged ovarian TME, cancer cells exhibit increased succinate production. Elevated extracellular succinate activates the SUCNR1 receptor on Tregs, promoting their recruitment and contributing to an immunosuppressive environment. This leads to diminished anti‐tumor T cell responses and reduced survival. Pharmacological inhibition of succinate reduces succinate levels, leading to decreased Treg recruitment, reduced immunosuppression, enhanced T cell mediated immunity, and improved survival outcomes.

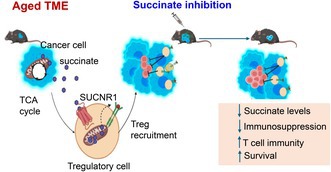

## 1 Introduction

Epithelial ovarian cancer (EOC), the most lethal gynecologic cancer, is a disease of older women, with a median age of 63 years at diagnosis (Langmar and Csomor 2006, 59). Despite breakthroughs in therapeutic options, EOC's prognosis remains dismal, with a survival of approximately 50% overall and approximately 20% for patients diagnosed at late stages (stage III/IV) (Torre et al. 2018, 181). Age is a known risk and prognostic factor in EOC, and survival outcomes are worse in older patients than in relatively younger patients, which is further influenced by treatment disparity and severe chemotherapy toxicity (Deng et al. 2017, 60; Tew 2016, 62; Won et al. 2013, 61). A recent epidemiologic study reported the median survival for women with EOC over the age of 65 years is 37.4 months, whereas it is 47.6 months for women under 65 years (Deng et al. 2017, 60). According to the SEER database, the survival rate for women with EOC is 42% when diagnosed above 65 years while it is 63.7% when diagnosed between 50 and 65 years, and 73.2% when diagnosed below 50 years of age (National Cancer Institute and Surveillance Epidemiology and End Results Program 2023). Despite this strong correlation, the molecular pathways that explain aging's negative impacts on EOC progression are unclear.

Aging is a hallmark of many diseases, including cancer (Anisimov 2007, 65; Ershler and Longo 1997, 66) attributed to the predominance of inflammation and associated immune decline (Caruso et al. 2004, 67; Foster et al. 2011, 68). With age, the accumulation of DNA damage and genetic mutations resulting from endogenous and exogenous insults leading to genomic instability (Hoeijmakers 2009, 356), can activate oncogenes or inactivate tumor suppressor genes, thereby promoting malignant transformation (Ou and Schumacher 2018, 357). Aging reshapes both the innate and adaptive immune systems, resulting in a decline in overall immune competence (Moskalev et al. 2020, 275), contributing to higher susceptibility to cancer. Aging related remodeling of the immune system is called immunosenescence, and is attributed to changes in lymphoid organs, cytokines and a shift in the relative abundance of T‐cell subsets (Rodriguez et al. 2020, 411). In this context, aging has recently been associated with increased immunosuppressive regulatory T cells (Tregs; CD4+ CD25+ FOXP3+), which suppress the number and function of antitumor T cells (Churov et al. 2020, 72; Garg et al. 2014, 276; Jagger et al. 2014, 71). Tregs suppress immune response via various mechanisms, including production of anti‐inflammatory cytokines like IL‐10 and TGFβ, by direct cell‐to‐cell interactions, upregulation of checkpoint proteins (Gonzalez‐Navajas et al. 2021, 271; Huang et al. 2015, 324; Kamada et al. 2019, 322; Kim et al. 2020, 272) and metabolic disruption of T cells (Li et al. 2020, 267; Zong et al. 2024, 266). It plays a key role in EOC (Yigit et al. 2010, 265), as their presence in tumor and circulation limits the antitumor activity of effector CD4^+^ and CD8^+^ T cells and other immune cells (Cassar et al. 2022, 29; Zhang et al. 2015, 264), resulting in poor prognosis (Kolben et al. 2022, 325; Li, Xu, et al. 2023; Li, Li, et al. 2023; Li, Guaman Tipan, et al. 2023, 327; Ye et al. 2020, 326). Previous reports have demonstrated Tregs to increase immunosuppression in aged tumors and play a crucial role in regulating the proliferation of effector T cells (Garg et al. 2014, 70) in melanoma and colon cancer (Chen et al. 2024, 296; Hurez et al. 2012, 328). This age‐related increase in Treg activity may contribute to even greater immune suppression, making immunotherapy less effective in this demographic. Cellular metabolism directs Tregs survival, proliferation, and their suppressive ability (He et al. 2017, 274). In contrast to antitumor effector CD4^+^ and CD8^+^ T cells, which primarily rely on glycolysis for their functions (Chang et al. 2013, 86; Liu, Zhang, et al. 2023; Liu, Liao, et al. 2023, 209; Reina‐Campos et al. 2021, 87), Tregs exhibit a distinct preference for oxidative phosphorylation (OXPHOS) (So et al. 2023, 74) for energy, differentiation and expression of anti‐inflammatory cytokines, like IL‐10 and TGFβ (Chavez and Tse 2021, 88; Moreno Ayala et al. 2019, 89). This reliance on OXPHOS allows Tregs to effectively modulate immune responses and maintain immune system homeostasis under normal conditions and in cancer. Thus, understanding the distinct metabolic pathways of the immune cells are crucial for developing targeted therapies aimed at enhancing antitumor immunity. Limited preclinical studies demonstrating the negative impact of age on EOC have revealed underlying mechanisms involving accumulation of senescent cells (Harper et al. 2022, 321), alteration of B cell related pathways in gonadal adipose tissue (Loughran et al. 2018, 243), and ultrastructural changes in collagen of aged omentum (Harper et al. 2022, 321). Our findings validated the rapid and aggressive development of EOC in aged mice as reported by others (Harper et al. 2018, 320; Loughran et al. 2018, 118; Sia et al. 2023, 318). We hypothesize that aging promotes the metabolic reprogramming of Tregs within the ovarian tumor microenvironment, enhancing their immunosuppressive function, impairing effector T cell responses, and thereby accelerating EOC progression in aged mice.

## 2 Materials and Methods

### 2.1 Cell Lines and Reagents

The mouse ovarian surface epithelial cancer cell line ID8^p53−/−^ and ID8^BRCA 1−/−^ was a kind gift from Dr. Ian McNeish (Ovarian Cancer Action Research Center, London, UK) (Walton et al. 2016, 90). The mouse ID8^p53+/+^, transduced with a lentiviral vector expressing firefly luciferase (ID8‐luc2), was kindly provided by Dr. John Liao, University of Washington, Seattle, WA (Liao et al. 2015, 233). The cell lines were maintained in Roswell Park Memorial Institute (RPMI) media (HyClone, Thermo Fisher Scientific, Waltham, MA), supplemented with 10% fetal bovine serum (BioAbChem, Ladson, SC). For in vitro succinate treatments, dimethyl succinate, a cell permeable succinate compound, was purchased from MilliporeSigma (Burlington, VT) (Ehinger et al. 2016, 310); CPI‐613 was a generous gift from Cornerstone Pharmaceuticals (Cranbury, NJ). (S)‐2‐[(2,6‐dichlorobenzoyl) amino] succinic acid (AA6) was purchased from MedChem Express (Monmouth Junction, NJ).

### 2.2 Mice

Female C57BL/6 mice aged 3 months (young/adult) and 22 months (old), representing ~25 and ~65 human years (Dutta and Sengupta 2016, 234), were obtained from Jackson Laboratory (Bar Harbor, ME) and housed under standard conditions at Henry Ford Hospital. All procedures followed institutional animal care and use committee approval (1587). Mice were acclimated for 1 week prior to experiments.

### 2.3 Tumor Induction and Monitoring

Mice were injected intraperitoneally (IP) with ID8‐luc2, ID8^p53−/−^ and ID8^BRCA 1−/−^ cells (5 × 10^6^ cells/200 μL) Phosphate buffered saline (PBS). Mice were weighed weekly. ID8‐luc2 mice underwent bioluminescence imaging weekly as described before using the Xenogen IVIS system 2000 series (Udumula et al. 2021, 56) (Perkin Elmer, Akron, OH). Bioluminescence signals were measured and quantified as total photon flux emission (photons/s) at the same exposure time for all mice using Living Image software (Perkin Elmer). For ID8^p53−/−^ and ID8^BRCA 1−/−^ mice, tumor burden was monitored by measuring body weight, abdominal circumference, and ascites formation weekly. Mice exhibiting severe clinical distress, including cachexia, anorexia, labored respiration, excessive ascites accumulation indicated by an abdominal circumference exceeding 8 cm, and those with impaired mobility or compromised bodily functions, were promptly euthanized to ensure ethical compliance and animal welfare (Udumula et al. 2021, 56; Udumula et al. 2023, 23). For CD25^+^ T cell depletion, mice with tumors received two IP injections of monoclonal anti‐mouse CD25 (IL‐2Rα) antibody (100 μg/200 μL PBS; clone PC‐61.5.3; BioXcell) (Lebanon, NH) twice weekly (Goschl et al. 2018, 98). Control mice received anti‐horseradish peroxidase immunoglobulin G1 isotype control (BioXcell). Depletion was evaluated by flow cytometry quantification of peripheral blood after 6 injections. For survival studies, mice were euthanized when the abdominal circumference reached 8 cm, as per the endpoint criteria approved by the hospital institutional animal care and use committee (protocol‐1587). At this stage, mice were humanely euthanized. Mice treated with AA6 received 12.5 mg/kg via IP injection thrice weekly for 4 weeks (Atlante et al. 2018, 337).

### 2.4 Immune Profiling

Tumor single cell suspension was prepared by dissociating tumor cells with Accutase at 37°C for 25 min on a shaking platform (Reichard and Asosingh 2019, 235) (STEMCELL Technologies, Cambridge, MA). The tumor suspension was filtered via a cell strainer and gently washed. The cell pellet was processed for flow cytometry analysis. Lymphocytes from tumors, ascites, and blood were also processed for surface marker staining (CD3, CD4, CD8, CD25, CD28, CD57, KLRG1, βgalactosidase, and PD1) and intracellular markers (FOXP3, IFNγ, Tbet, Granzyme B, Perforin, IL‐10, γH2AX, RAD51, and TGFβ) as before (Udumula et al. 2021, 56; Udumula et al. 2023, 23). Flow cytometric analysis was performed on Attune NxT (Thermo Fisher Scientific), and results were analyzed using FlowJo software (Version 10.9.0; FlowJo LLC, Ashland, OR). T‐Distributed Stochastic Neighbor Embedding clusters were prepared using FlowJo (Version 10.9.0) (Udumula et al. 2023, 23; Zahoor et al. 2025, 215). All fluorochrome antibodies were from BioLegend (San Diego, CA) and listed in Table 1.

**TABLE 1:** List of flow antibodies.

Reagent	Source	Identifier
CD45	S18009F	Biolegend
CD3	17A2	Biolegend
CD4	GK.1.5	Biolegend
CD8	53–6.7	Biolegend
CD25	PC61	Biolegend
PD1	RMP1‐30	Biolegend
IFNγ	XMG1.2	Biolegend
IL‐10	JES5‐16E3	Biolegend
TGF‐β	TW7‐16B4	Biolegend
FOXP3	MF‐14	Biolegend
Granzyme B	QA16A02	Biolegend
Perforin	S16009A	Biolegend
Tbet	4B10	Biolegend
CD28	37.51	Biolegend
CD57	QA17A04	Biolegend
KLRG1	2F1/KLRG1	Biolegend
βgalactosidase	40‐1a	Santacruz

### 2.5 T Cell Suppression Assay

Splenic CD4^+^ T and CD8^+^ T cells were isolated from young mice (more than 92% purity) using a MojoSort CD4^+^ T cell and CD8^+^ T cell isolation kit (BioLegend), labeled with carboxyfluorescein diacetate, succinimidyl ester; 5 mM, as per the manufacturer. Splenic CD4+ CD25+ FOXP3+ cells were isolated using a MojoSort Isolation Kit (BioLegend) (> 94% purity). CD4^+^ T cells and CD8^+^ T were co‐cultured with Treg cells at a ratio of 2:1 in 200 μL of T cell assay media containing CD3/CD28 Dyna beads and recombinant mouse IL‐2 (20 U/mL). After 72 h, flow cytometry was performed for measuring CD4^+^ T cell and CD8^+^ T cell proliferation (Chen et al. 2005, 237; Dowling et al. 2018, 236; Geels et al. 2024, 238).

### 2.6 Quantitation of Metabolites by Liquid Chromatography‐Tandem Mass Spectroscopy

Tricarboxylic acid cycle standard mix 1 and 2 (cat no. MSK‐TCA1 and MSK‐TCA 2 A) and its 13C‐labeled metabolites were purchased from Cambridge Isotope Laboratories Inc. (Tewksbury, MA). Acetonitrile (High Performance Liquid Chromatography grade), formic acid, Mass Spectrometry grade water, and methanol were purchased from Sigma‐Aldrich (Burlington, VT). The levels of TCA metabolites in various samples were quantified by ultrahigh performance liquid chromatography‐tandem mass spectroscopy (Waters, Milford, MA) as described before (Udumula et al. 2024, 213). Glycolysis metabolomic profiling was performed by Metabolon Inc. (Morrisville, MA) (Udumula et al. 2023, 23). Young and old control serum was purchased from George King Bio‐Medical (Overland Park, KS).

### 2.7 Seahorse Metabolic Phenotype

Splenic Tregs from young and aged control and ID8^p53−/−^ EOC mice were plated (5 × 10^5^ cells/well) on Cell‐Tak coated XFe96 plates, and Oxygen Consumption Rate and Extracellular Acidification Rate were measured using the Agilent Seahorse XFe96 Analyzer as published earlier (Udumula et al. 2024, 213; Udumula et al. 2021, 56; Udumula et al. 2023, 23).

### 2.8 Single Cell ENergetIc Metabolism by profilIng Translation inHibition (SCENITH)

Briefly, freshly isolated splenocytes from ID8^P53−/−^ young and old mice were resuspended in complete RPMI at 1–2 × 10^6^ cells/mL and divided into four conditions: (i) untreated control, (ii) oligomycin (1 μM) to inhibit mitochondrial ATP synthase, (iii) 2‐deoxy‐D‐glucose (2DG; 50 mM) to inhibit glycolysis, and (iv) oligomycin plus 2DG. Cells were incubated with the respective inhibitors for 30 min at 37°C, followed by the addition of puromycin (10 μg/mL) for 30 min to label newly synthesized proteins. Cells were then washed, surface‐stained for lineage markers, fixed, permeabilized, and stained intracellularly with anti‐puromycin antibody. Samples were acquired by flow cytometry, and puromycin mean fluorescence intensity (MFI) was quantified in defined immune cell subsets (Zahoor et al. 2025, 215). Relative dependence on glycolysis and oxidative phosphorylation was calculated by comparing puromycin incorporation across inhibitor conditions as a measure of protein synthesis under pathway restriction (Arguello et al. 2020, 316).

### 2.9 Western Blot

Total protein was isolated, quantified, and separated by 10%–12% SDS polyacrylamide gel electrophoresis followed by immunoblotting with γH2AX, RAD51, and SUCNR1 antibody (Thermo Fisher Scientific as before) (Mangali et al. 2019, 309; Udumula et al. 2022, 102).

### 2.10 Quantitative‐PCR

Total RNA was isolated using the RNeasy kit (Qiagen) (Redwood city, CA), and cDNA was synthesized by iScript cDNA synthesis kit (Bio‐Rad) (Hercules, CA) according to the manufacturer's guidelines. Quantitative PCR analysis was performed using Bio‐Rad CFX96 real‐time PCR detection system (CFX Maestro, Bio‐Rad) as before with ribosomal L27 as housekeeping gene (Udumula et al. 2024, 213; Udumula et al. 2023, 23). Primers' sequences are listed in Table 2.

**TABLE 2:** List of primers.

Primers	Sequence
SDHa	**F** GAG ATG TGG TGT CTC GGT CCA T **R** GCT GTC TCT GAA ATG CCA GGC A
SDHb	**F** GCA GTC CAT AGA AGA GCG TGA G **R** TGT CTC CGT TCC ACC AGT AGC T
SDHc	**F** CCTACTCTCGGCCTAGAAGC **R** GTCCTTTAAGCCAGCAACCC
L27	Real time primers

*Note:* Bold is used to refer to Forward and reverse primer sequences.

### 2.11 Patient Serum Studies

Patient blood was collected prior to surgery under an approved institutional review board protocol (# 9520), following informed patient consent and conducted in compliance with the approved methods, guidelines, and regulations. Serum was isolated from the blood of patients under 50 years (young cohort, *n* = 5) and over 65 years of age (older cohort, *n* = 5), diluted in complete RPMI media at a 1:25 ratio and then exposed to splenic Treg cells isolated from naive mice. After 48 h, the Tregs were analyzed for expression of FOXP3, IL‐10, and TGFβ by flow cytometry as published before (Udumula et al. 2021, 56; Udumula et al. 2023, 23). For succinate treatments, splenic Treg cell cultures were treated with succinate (100 μM, 1, and 5 mM) for 48 h followed by flow cytometry analysis.

### 2.12 5‐Ethynyl‐2′‐Deoxyuridine Staining for Proliferation

An EdU Proliferation Assay Kit (Click‐iT EdU Cell Proliferation Kit for Imaging, Alexa Fluor 594 dye, Thermo Fisher Scientific) (Chapman et al. 2012, 212) was used to detect Treg cell proliferation. In brief, 5 × 10^5^ splenic Tregs isolated from naïve mice were cultured on cover slips in 24‐well plates overnight in the presence of young or old ascites along with the culture medium supplemented with 20 mM EdU reagent. After 48 h, cells were fixed with paraformaldehyde and stained with DAPI. EdU‐positive cells were analyzed and quantified by time‐lapse fluorescence microscopy (Lonheart FX Automated Microscope, Winooski, VT) and ATTUNE NXT Flow cytometer (Thermo Fisher Scientific).

### 2.13 ELISA

IL‐6, IL‐1β, IL‐4, IL‐10, IFNγ, MCP‐1 and TNFα levels were measured by ELISA kits from BioLegend. Insulin, IGF‐1, Leptin, adiponectin, and GMCSF were measured by kits from R&D Systems (Minneapolis, MN), as per manufacturers' instructions and as published before (Udumula et al. 2023, 23).

### 2.14 Succinate Dehydrogenase Assay

Succinate dehydrogenase assay (SDH) activity in splenic Tregs was measured using a colorimetric assay (Abcam) per manufacturer instructions (Almohaimeed et al. 2022, 239).

### 2.15 Statistical Analysis

Statistical tests were performed using GraphPad Prism 8 (Dotmatics, Boston, MA). Data were analyzed by unpaired *t*‐test or one‐way analysis of variance as appropriate. Survival curves were evaluated by Kaplan–Meier analysis with Mantel‐Cox and Gehan‐Breslow‐Wilcoxon tests. Significance was defined as *p* < 0.05.

## 3 Results

### 3.1 Aging Exacerbates Preclinical EOC Models

**FIGURE 1:**
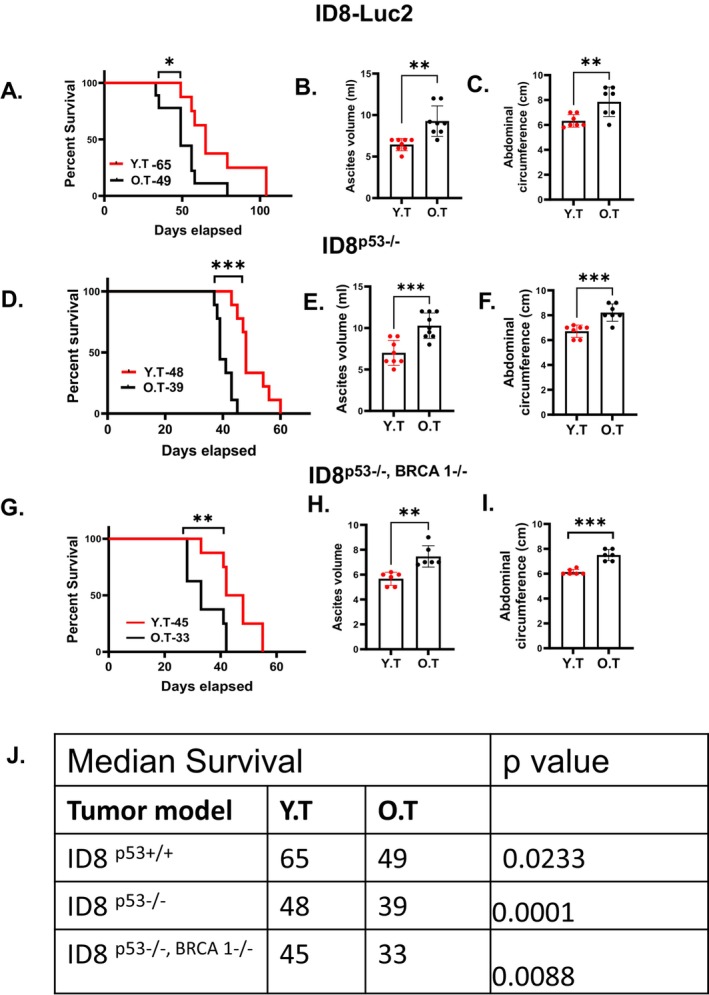
Aging exacerbates in preclinical EOC models: Kaplan–Meier graphs indicating overall survival in mice bearing (A–I) (A) ID8‐luc2 EOC injected in Young Tumor (YT) or Old Tumor (OT) (*n* = 10/group) mice, *p* = 0.0233 by Gehan‐Breslow‐Wilcoxon test; (D) ID8p53−/− EOC injected in YT or OT (*n* = 10/group), *p* < 0.0001 by Gehan‐Breslow‐Wilcox test and (G) ID8p53−/−, BRCA 1−/− EOC injected in YT or OT (*n* = 10/group), *p* = 0.0088 by Gehan‐Breslow‐Wilcoxon test. (B, E, and H) Ascites volume collected at 6 weeks from ID8‐luc2, and at 5 weeks from ID8p53−/− and ID8p53−/−, BRCA 1−/− EOC bearing young and old mice. (C, F, I) Bar graph represents average abdominal circumference at 6 weeks of ID8‐luc2, and at 5 weeks of ID8p53−/− and ID8p53−/−, BRCA 1−/− EOC bearing young and old mice. (J) The table represents median survival in all three mouse models of young and old EOC mice. *p < 0.01, ***p < 0.001, OT compared with YT group by Student's *t*‐test.

To understand aging's impact on EOC, we compared the tumor progression and survival of IP injected mouse ID8 EOC cells with different gene signatures including ID8^p53+/+^‐luc2, ID8^p53−/−^ and ID8^p53−/−,BRCA 1−/−^ in young and old C57BL/6 female mice (Roby et al. 2000, 216; Walton et al. 2016, 90). Old mice bearing ID8‐luc2 tumors had shorter median survival (49 vs. 65 days in young mice) (Figure 1A,J) and showed greater tumor burden, confirmed by increased abdominal circumference, ascites (Figure 1B,C), and higher bioluminescence imaging signals (Figure S1A,B). The mice with ID8^p53−/−^ and ID8^p53−/−,BRCA 1−/−^ tumors representing high grade serous ovarian cancer (HGSOC) (Mandilaras et al. 2019, 218) showed similar patterns. Old mice with ID8^p53−/−^ tumors had a median survival of 39 days compared to 48 days in young mice (Figure 1D,J), along with increased abdominal circumference and ascites accumulation (Figure 1E,F). Old mice with ID8^p53−/−,BRCA 1−/−^ had a median survival of 33 days compared to 45 days in young mice (Figure 1G,J), and an increased abdominal circumference and ascites volume (Figure 1H,I). These observations are in accordance with other reports (Hou et al. 2022, 11; Loughran et al. 2018, 118; Shim et al. 2022, 244) that demonstrate similar aggravated EOC progression in older mice, thus, confirming aging exacerbates EOC progression. Aging is also associated with increased levels of cellular senescence and inflammatory factors (Bottazzi et al. 2018, 219; Li, Xu, et al. 2023; Li, Li, et al. 2023; Li, Guaman Tipan, et al. 2023, 220), defined as senescence‐associated secretory phenotype (SASP) that support a tumor permissive microenvironment (Dong et al. 2024, 254; Kuilman et al. 2008, 255; Narita and Lowe 2005, 253). In accordance, we observed metabolic growth factors IGF‐1 (Figure S2B,F) and leptin (Figure S2D,H) levels were significantly increased in the ascites of aged mice compared to young mice bearing ID8^p53−/−^ and ID8 ^p53−/−,BRCA1−/−^ tumors, while insulin (Figure S2A,E) was lower in the aged ID8^p53−/−^ mice compared to young mice but was unchanged in ID8^p53−/−,BRCA1−/−^ mice.

Adiponectin levels were unchanged in the ascites of ID8^p53−/−^ mice but reduced in aged ID8 ^p53−/−,BRCA1−/−^ mice relative to younger mice (Figure S2C,G). Among SASP markers, levels of IL‐4 (Figure S2I,M), IL‐10 (Figure S2K,O), MCP‐1 (Figure S2J,N), IL‐1β (Figure S2Q,U), IL‐6 (Figure S2S,W), and TNFα (Figure S2R,V) were significantly increased in ascites of aged ID8^p53−/−^ and ID8 ^p53−/−,BRCA1−/−^ mice, compared to young mice. In contrast, GMCSF levels were decreased in both aged models (Figure S2L,P). IFNγ, a key cytokine for cytotoxic T cells, was lower in the ascites of aged mice of ID8^p53−/−^ (Figure S2T) while it was increased in ID8 ^p53−/−,BRCA 1−/−^ mice compared to young EOC mice (Figure S2X). These findings are consistent with other cancer studies indicating that the aging Tumor microenvironment (TME) induces distinct modifications that contribute to the establishment of a pro‐tumorigenic milieu (Fane and Weeraratna 2020, 189; Hibino et al. 2021, 257).

### 3.2 Aging Induces Differential Systemic T Cell Response in EOC

Age‐related immune decline, particularly in T cell composition, is well‐established (Salam et al. 2013, 353; Yager et al. 2008, 354). To examine this, we profiled major immune populations in the blood of young tumor‐bearing mice, old tumor‐bearing mice, young, and old control mice without tumors. In control old mice without tumors, we found no significant changes in the number of CD4^+^ T cells, while the CD8^+^ T cell number was significantly decreased and a notably higher prevalence of Tregs was observed in old control mice compared to young controls (Figure S3C,F,E), consistent with previous studies (Deng et al. 2017, 60; Garrido‐Rodriguez et al. 2021, 370; Li et al. 2019, 369; Palatella et al. 2022, 277; Yang et al. 2024, 262). In EOC‐bearing mice, the young mice exhibited an increase in the number of both CD4^+^ and CD8^+^ T cells compared to young control mice without tumors (Figure S3A–C,F), along with a robust increase in effector CD4+ IFNγ+ (Figure S3D) and CD8+ IFNγ+ cells (Figure S3G). Additionally, cytotoxic markers, granzyme B (Grz b) and perforin, were significantly increased in young mice with tumors, highlighting an effective T cell‐mediated response against the tumor (Figure S3H,I). In contrast, old mice with EOC showed a marked decline in both CD4^+^ and CD8^+^ T cells compared to old controls without tumors (Figure S3A–C,F). The failure to mount a comparable antitumor immune response by old EOC mice was reflected in decreased effector CD4+ IFNγ+ and CD8+ IFNγ+ cells, and cytotoxic CD8+ Grz b+ and CD8+ perforin+ cells compared to young EOC mice (Figure S3D,G,H,I). While Tregs were increased in both young and old EOC mice, it was most pronounced in old EOC mice (Figure S3A,B,E), suggesting a heightened Treg‐mediated immunosuppression. Analysis of control mice revealed that the percentage of total macrophages (CD11b^+^F4/80^+^) was lower in old mice compared to young counterparts (Figure S3J).

Additionally, there were no significant changes in the expression of M1 (CD38) (Figure S3K) or M2 markers (EGR2 and CD206) between young and old control mice (Figure S3L,M). The co‐expression of CD38/EGR2 and CD38/CD206 also remained comparable between the groups (Figure S3N,O). Notably, the M1 macrophage marker iNOS was significantly reduced in aged control mice relative to young controls (Figure S3P). Intracellular M2 markers (EGR2^+^Arg1^+^ and CD206^+^Arg1^+^), as well as myeloid‐derived suppressor cells (CD11b^+^Gr1^+^), did not differ significantly between young and aged control mice (Figure S3Q–S). No significant increase in the percentage of F4/80^+^ cells was observed between young EOC‐bearing mice and young control mice. In contrast, a significant increase in F4/80^+^ cells was detected in old EOC‐bearing mice compared to aged control mice (Figure S3J). The percentage of F4/80+ CD38+ cells did not differ significantly between young and old EOC mice compared to their respective young and old control groups (Figure S3K). The percentage of F4/80+ EGR2+ cells remained unchanged between young EOC mice and young control mice; however, a significant increase in F4/80+ EGR2+ cells was observed in old EOC mice compared to both young EOC and old control mice (Figure S3L). Both young and old EOC mice exhibited an increased frequency of CD206+ and CD11b+ Gr1+ populations compared to their age‐matched control mice; however, no significant differences were observed between the young and old EOC groups (Figure S3M,S). Additionally, a decrease in the M1/M2 ratio (CD38/EGR2) was observed in older EOC mice compared to young EOC mice, although this ratio did not differ significantly when compared to other groups (Figure S3N). The overall M1/M2 ratio (CD38/CD206) was reduced in EOC mice of both age groups relative to their non‐tumor‐bearing counterparts (Figure S3O), indicating an increased presence of M2‐like macrophages associated with tumor progression.

Additionally, the M1 macrophage marker iNOS showed a significant decline in both young and old EOC mice compared to young control mice, with the decrease particularly pronounced in the old EOC group (Figure S3P). This suggests a diminished pro‐inflammatory, classically activated macrophage response in the presence of EOC, especially with aging. Interestingly, despite this decline, the levels of CD38+ iNOS+ macrophages were significantly elevated in old EOC mice compared to their age‐matched non‐tumor controls, indicating a complex regulation of M1 macrophage subsets within the aged tumor microenvironment. On the other hand, the intracellular M2 marker Arginase 1 (EGR2+ Arg1+) did not show significant changes across the groups (Figure S3Q). These findings suggest that aging impacts macrophage polarization more in tumor‐bearing mice, with older mice showing a shift towards a more immunosuppressive M2 phenotype. While macrophage numbers are not significantly reduced in either young or old tumor‐bearing mice, the M1/M2 balance shifts in older mice, which may contribute to a less effective immune response against EOC. This data suggests that compromised macrophage function, along with increased immunosuppressive Tregs, exacerbates the impaired anti‐immune response in old mice, potentially contributing to rapid progression of EOC in older mice.

### 3.3 Aging Diminishes the Intratumor T Cell Response in EOC

**FIGURE 2:**
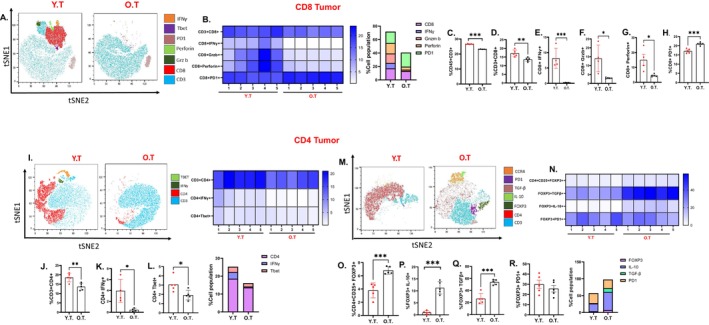
Aging diminishes the intra‐tumor T cell response in EOC: ID8p53−/− EOC injected in YT or OT (*n* = 10/group), Immune profiling was performed in tumors of 5 individual mice per group (A–R) (A) A representative t‐SNE visualization of markers after gating on single, live, CD45+ CD3+, CD4+, CD8+, IFNγ, Granzyme B, perforin and PD1 expression. (B) Heatmap represents marker expression of the main T cell subsets in individual samples. Bar plots represent the percentage of T cell subsets (C) CD45+ CD3+ (D) CD8+, (E) CD8+ IFNγ+, (F) CD8+ GrzB+, (G) CD8+ perforin, (H) CD8+ PD1. (I) A representative t‐SNE visualization of markers after gating on single, live, CD45+ CD3+, CD4+, IFNγ, and Tbet expression. Bar plots represent (J) CD4+, (K) CD4+ IFNγ+ (L) CD4+ Tbet. (M) A representative t‐SNE visualization of markers after gating on single, live, CD45+ CD3+, CD4+, CD25+, FOXP3, IL‐10, TGF‐β and PD1 expression. (N) Heatmap represents marker expression of the main T cell subsets in 5 individual samples. Bar plots represent the percentage of T cell subsets (O) FOXP3+, (P) FOXP3+ IL‐10, (Q) FOXP3+ TGFβ, (R) FOXP3+ PD1 in the tumors. The experiment was repeated twice in two different sets of mouse experiments. *p < 0.05, **p < 0.01, ***p < 0.001, OT compared to YT group by Student's *t*‐test.

To validate that the age associated dampened systemic antitumor immune response was mirrored in tumors and TME, we characterized major T cell subsets in the tumors and ascites of young and old mice with ID8^p53−/−^ and ID8^p53−/−,BRCA 1−/−^ tumors. ID8^p53−/−^ tumors of the old mice showed a significant decrease in the overall number of CD8^+^ T cells (Figure 2A–D), with a marked reduction in the intracellular expression of key effector and cytotoxic markers, including IFNγ, Grz b+, and perforin (Figure 2E–G), while the exhaustion marker PD‐1 was increased (Figure 2H) compared to young EOC mice. Similarly, a significant decrease in overall CD4^+^ T cells (Figure 2I,J) along with a reduction in effector CD4+ IFNγ+ and CD4+ Tbet+ cells (Figure 2K,L) was observed in tumors from old mice compared to young mice. Most impressively, tumors from old mice demonstrated a significant increase in the overall number of Tregs (Figure 2M–O), and Tregs expressing suppressive cytokines IL‐10 and TGFβ (Figure 2P,Q), while PD1 expression was unchanged compared to tumors from young mice (Figure 2R). An identical pattern was observed in the ascites, where we noted a decrease in both CD4^+^ and CD8^+^ T cell frequency and the intracellular effector markers (Figure S4A–L), with a concurrent increase in Tregs frequency and IL‐10 and TGFβ (Figure S4M–Q). Unlike the intratumoral Tregs, we observed increased PD1 expression in Tregs from ascites (Figure S4R). These findings point to an enhanced immunosuppressive Treg response in the tumors and ascites of old mice with EOC, which may further hinder effective antitumor immunity.

In the ID8p53 ^−^/^−^; BRCA1 ^−^/^−^ EOC model, aging did not significantly alter the overall frequency of CD4 ^+^ or CD8 ^+^ T cells within tumors (Figure S5B,H). However, aged tumors exhibited a marked decline in effector T cell function, as evidenced by reduced frequencies of CD4 ^+^ IFNγ ^+^, CD4 ^+^ T‐bet^+^, CD8 ^+^ IFNγ ^+^, CD8 ^+^granzyme B^+^, and CD8 ^+^perforin^+^ cells (Figure S5C,D,I–K). In contrast, the proportion of Tregs was significantly increased in aged tumors, accompanied by elevated expression of IL‐10 and TGFβ, indicating enhanced immunosuppressive activity (Figure S5E–G). Similar trends were observed in the ascitic compartment, where aging was associated with reduced effector CD4 ^+^ and CD8 ^+^ T cell responses and increased Treg associated suppressive markers (Figure S6). Together, these findings indicate that aging primarily impairs the functional capacity of tumor‐infiltrating T cells while promoting an immunosuppressive Treg environment in EOC.

### 3.4 Tregs From Old Mice Exhibit Increased Immunosuppressive Ability

**FIGURE 3:**
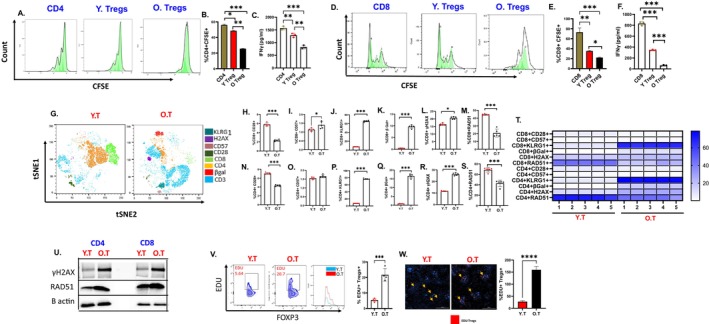
Tregs from old mice are more immunosuppressive: Naïve CD4 and CD8 T cells were isolated and were labeled with CFSE for 20 mins. These cells were co cultured with Tregs from young and old ID8^p53−/−^ EOC mice at a ratio of 2:1. (A) CFSE proliferation histograms representing CD4+ proliferation in presence of Tregs from old and young EOC mice. (B) Bar graph represents %CD4+ CFSE. (C) Bar graph represents IFNγ levels from CD4: Treg co‐culture experiment. (D) CFSE proliferation histograms representing CD8 proliferation in presence of Tregs from old and young EOC mice. (E) Bar graph represents %CD8+ CFSE. (F) Bar graph represents IFNγ levels from CD8: Treg co‐culture experiment. (G) A representative t‐SNE visualization of markers after gating on single, live, CD45+ CD3+, CD4+, CD8+, CD28, CD57, H2AX, βGal and KLRG. Bar plots represent the percentage of senescence markers on T cell subsets (H) CD8+ CD28, (I) CD8+ CD57, (J) CD8+ KLRG1, (K) CD8+ βGal, (L) CD8+ H2AX, (M) CD8+ RAD51, (N) CD4+ CD28, (O) CD4+ CD57, (P) CD4+ KLRG, (Q) CD4+ βGal, (R) CD4+ γH2AX and (S) CD4+ RAD51. (T) Heatmap represents marker expression of the main senescence markers on T cell subsets in individual samples. (U) Immunoblot expression of γH2AX and RAD51 from CD4 and CD8 T cells isolated from spleens of young and old EOC mice. (V) Flow plots and bar graph representing %FOXP3+ EDU. (W) Immunocytochemistry images representing EDU+ Tregs in red. *p < 0.05, **p < 0.01, ***p < 0.001, OT compared to YT group by Student's *t*‐test.

Since the most significant changes were observed in Tregs, which inhibit T cell proliferation and effector activity (Chen et al. 2005, 222; Schmidt et al. 2012, 221), we assessed the immunosuppressive ability of Tregs from old and young mice. We co‐cultured splenic Tregs isolated from young and old EOC mice with CFSE labeled naïve CD4+ and CD8+ T cells from young mice at a 1:2 ratio in the presence of IL‐2 (20 ng/mL) for 72 h. Flow cytometric analysis showed that Tregs from old EOC mice inhibited the proliferation of both CD4+ and CD8+ T cells to a greater extent compared to Tregs from young EOC mice (Figure 3A,B,D,E) and were particularly effective in inhibiting the production of IFNγ by both CD4+ and CD8+ T cells (Figure 3C,F). In the same co‐culture experiment, we profiled key senescence markers on CD4+ and CD8+ T cells and observed a significant reduction in CD28 percentage (Figure 3H,N,T), a co‐stimulatory molecule essential for T cell activation (Effros 1997), in the presence of Tregs derived from old EOC mice compared to young EOC mice. Additionally, CD57, a marker associated with terminally differentiated or senescent T cells with diminished proliferative capacity (Cura Daball et al. 2018, 224), was significantly increased in both CD4+ and CD8+ T cells from aged EOC mice (Figure 3I,O,T). Other senescence markers, such as KLRG1 (Figure 3J,P,T) and β‐galactosidase (Figure 3K,Q,T), which reflect a permanent loss of proliferative potential (Liu et al. 2018, 379; Ramello et al. 2021, 225), were also significantly elevated in CD4+ and CD8+ T cells from aged EOC mice. To further evaluate whether T cells from aged EOC mice exhibit increased DNA damage, we examined the expression of γ‐H2AX, a marker of DNA double‐strand breaks, and RAD51, a key mediator of homologous recombination dependent DNA repair. Both γ‐H2AX and RAD51 levels were significantly elevated in CD4^+^ and CD8^+^ T cells from aged EOC mice compared with those from young controls, as demonstrated by flow cytometric analysis (Figure 3L–S) and Western blotting (Figure 3U). These findings indicate that T cells within aged tumors experience increased genomic stress and activation of DNA damage response pathways, which may contribute to their impaired functional capacity. Next to see if EOC TME can influence Tregs, we conducted a Click‐IT EdU proliferation assay on naive Tregs exposed to ascites isolated from EOC‐bearing young and old mice. Tregs exposed to ascites from old mice underwent increased proliferation, shown by increased EdU incorporation, compared to Tregs cultured in ascites from young mice (Figure 3V,W). Together, these observations show strong evidence that Tregs from old mice have enhanced suppressive function and promote T‐cell senescence as a suppressive mechanism (Liu et al. 2021, 250; Ye et al. 2012, 249), contributing to an increased immunosuppressive TME and potentially facilitate immune evasion by EOC.

### 3.5 Tregs Are Key in Promoting EOC in Aged Mice

To investigate if Tregs are crucial in promoting EOC progression in old mice, we utilized the anti‐CD25 (clone PC‐61) antibody to deplete Tregs in ID8^p53−/−^ tumor‐bearing old mice (Figure S7A) (Kalim et al. 2022, 251; Li, Xu, et al. 2023; Li, Li, et al. 2023; Li, Guaman Tipan, et al. 2023, 358). Depletion of Tregs significantly enhanced the survival of the old mice, with an increase in median survival to 64 days, compared to 45 days in the control antibody group (Figure S7B). The decreased tumor burden was reflected by low ascites accumulation in the depletion mice compared to control mice (Figure S7C). High dimensional immune profiling revealed substantial remodeling of the immune landscape following Treg depletion. tSNE analyses demonstrated a clear shift in the immune cell composition within tumors, ascites, and peripheral blood following Treg depletion (Figures S7D, S8A, and S9A). Quantitative analysis showed that Treg depletion significantly reduced the frequency of CD4^+^ CD25^+^ FOXP3^+^ Tregs, as well as immunosuppressive FOXP3^+^IL‐10^+^ and FOXP3^+^ TGF‐β^+^ subsets across tumor, ascites, and blood compartments (Figures S7G–I, S8D–F, and S9D–F) (Jarnicki et al. 2006, 366). In contrast, effector T‐cell responses were enhanced following Treg depletion. Within tumors, the frequency of CD3^+^CD4^+^ and CD3^+^CD8^+^ T cells increased, accompanied by elevated IFNγ‐producing CD4^+^ and CD8^+^ T cells, indicating improved effector function (Figure S7E,F,K,L). Similarly, ascites and peripheral blood analyses revealed increased CD8^+^ T‐cell frequencies and effector cytokine production, including IFNγ, perforin, and granzyme B, following Treg depletion (Figures S8H–K and S9H–K). These findings suggest that Tregs suppress systemic and local anti‐tumor immunity during EOC progression. We next assessed markers associated with T‐cell exhaustion and senescence. Treg depletion significantly reduced the frequency of CD8^+^ PD1^+^ T cells within tumors (Figure S7O), indicating a reduction in exhaustion phenotypes. Moreover, markers associated with T‐cell senescence were markedly decreased following Treg depletion. In tumor‐infiltrating CD8^+^ T cells, β‐galactosidase, KLRG1, and CD57 expression were reduced, while the co‐stimulatory molecule CD28 was restored (Figure S7P–W). Similar trends were observed in ascites and blood compartments, where Treg depletion significantly decreased senescence‐associated markers such as β‐galactosidase and KLRG1, while increasing CD28 expression in both CD4^+^ and CD8^+^ T cells (Figures S8M–S and S9M–T). Together, these findings underscore the pivotal role of Tregs in promoting EOC progression in aged mice, and absence of Tregs allows for a more robust immune response against EOC cells.

### 3.6 Tregs From Aged EOC Mice Prefer OXPHOS for Immunosuppression

**FIGURE 4:**
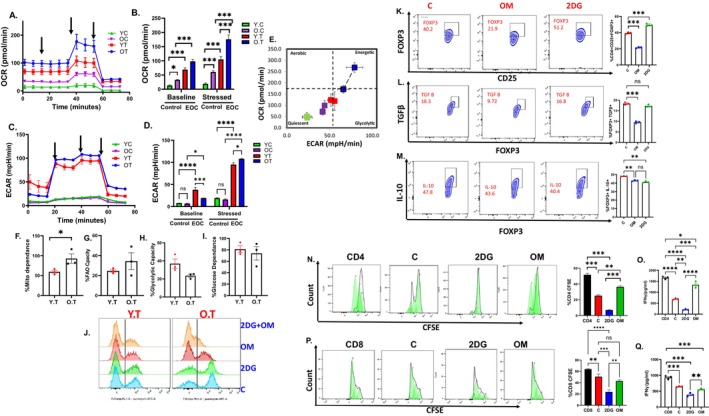
Tregs from aged EOC mice prefer OXPHOS for immunosuppression: ID8p53−/− EOC injected in YT or OT, FOXP3+ cells were isolated from the spleens of control and EOC young and old tumor at 5 weeks and subjected to Seahorse analysis and energy targeted metabolomics. (A) Oxygen consumption rate (OCR) was assessed in real‐time using an XFe96 Seahorse analyzer as described in methods. Port injections of (1) oligomycin, (2) FCCP, and a combination of (3) rotenone‐antimycin were given. (B) The bar graph represents basal and stressed OCR (*n* = 3). (C) Extracellular acidification rate (ECAR) was measured with port injections of (1) glucose, (2) oligomycin, and (3) 2‐DG. (D) The bar graph represents basal and stressed ECAR (*n* = 3). (E) A quadrant plot indicates the metabolic shift of energy phenotype at basal and stressed levels. SCENITH was performed in Tregs isolated from young and old p53−/− EOC mice. Bar graphs represent the (F) %Mitochondrial dependence, (G) % FAO Capacity, (H) %Glucose dependence, (I) Glycolytic capacity. (J) Histogram representing the FOXP3+ Puromycin in Tregs isolated from young and old EOC mice after treatment. Tregs isolated from young tumor bearing mice were treated with 2DG and oligomycin, (K) Flow plots and bar graph represent FOXP3 percentage. (L) Flow plots and bar graph represent TGFβ percentage. (M) Flow plots and bar graph represent IL‐10 percentage. (N) CFSE proliferation histograms and bar graph representing CD4 proliferation in presence of Tregs treated with 2DG and oligomycin. (O) Bar graph represents IFNγ levels from CD4: Treg co‐culture treated with 2DG and oligomycin. (P) CFSE proliferation histograms and bar graph representing CD8 proliferation in presence of Tregs treated with 2DG and oligomycin. (Q) Bar graph represents IFNγ levels from CD8: Treg co‐culture treated with 2DG and oligomycin. *p < 0.05, **p < 0.01, ***p < 0.001 tumor compared to control as assessed by unpaired *T*‐test.

T cell function, differentiation, and activation are largely governed by metabolic modifications (Kouidhi et al. 2017, 245; Ma et al. 2024, 246). Previous research has shown that Tregs to be dependent on OXPHOS for activation and function (Han, Peng, et al. 2023; Han, Georgiev, et al. 2023, 248; Yan et al. 2022, 114). Bioenergetics of splenic Tregs isolated from young and old mice with and without EOC were profiled by XFe96 Seahorse analyzer. At the baseline level, the Tregs from young mice without EOC displayed lower OXPHOS measured as OCR, which did not change even under stress. The Tregs from old mice with EOC showed significantly increased OXPHOS compared to Tregs from young tumor‐bearing mice, and both young and aged healthy controls, under both basal and stressed conditions (Figure 4A,B,E and Figure S10A,B). The basal ECAR, which reflects glycolytic activity, did not differ between young and old control or tumor‐bearing Tregs at basal level; however, old Tregs from EOC mice exhibited increased glycolysis under stressed conditions when compared to young Tregs from EOC mice and their controls (Figure 4C,D and Figure S10C,D). The resulting energy phenotype indicated a basal aerobic energy phenotype in the Tregs from old EOC mice that further moved to an energetic phenotype when stressed (Figure 4E). To validate the observations of splenic Tregs, we used SCENITH to profile the metabolic dependency of Tregs from ascites of young and old mice. Tregs from old EOC mice had higher mitochondrial dependency, decreased glycolytic capacity and non‐significant increase in fatty acid oxidation capability compared to Tregs from young EOC mice (Figure 4F–H). While no significant changes were observed in glucose dependency between young and old Tregs (Figure 4I). When Tregs were treated with OXPHOS inhibitor oligomycin, Tregs from old mice showed a decrease in expression of key immunosuppressive markers, including FOXP3, TGFβ and IL‐10 (Figure 4K–M), while treatment with the glycolysis inhibitor 2DG had no effect. Oligomycin treatment resulted in reversal of CD4^+^ T cell suppression, indicating a functional reprogramming of Treg cells (Figure 4N). This effect was observed in comparison to treatment with 2DG, which primarily targets glycolysis (Figure 4M). We also observed that inhibiting glycolysis led to a significant decrease in IFNγ production. Importantly, treatment with oligomycin reversed the suppression of IFNγ levels, further supporting the idea that glycolysis plays a crucial role in maintaining the effector functions of T cells (Figure 4O). Consistently, CD8^+^ T cells showed limited proliferation when co‐cultured with Tregs under control conditions. Glycolysis inhibition with 2DG partially restored CD8^+^ T‐cell proliferation, whereas OXPHOS inhibition with oligomycin resulted in a stronger recovery of proliferation (Figure 4P,Q). Together, these results indicate that OXPHOS‐dependent metabolic programming drives the suppressive function of Tregs in aged EOC, and targeting mitochondrial metabolism can restore anti‐tumor T cell responses.

#### 3.6.1 Succinate as a Metabolic Regulator of Tregs in Aged Mice With EOC

**FIGURE 5:**
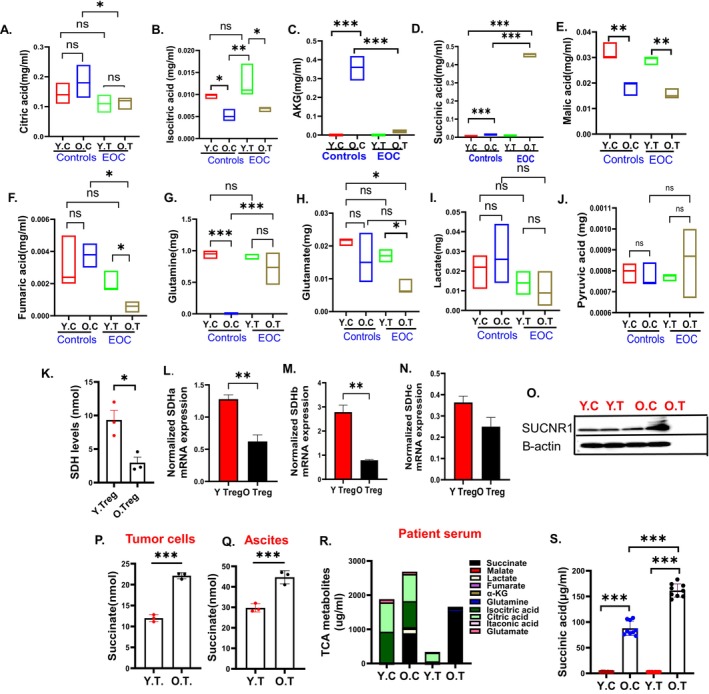
Succinate as a metabolic regulator of Tregs of aged mice with EOC: Tregs were isolated from spleens of control and ID8 ^p53−/−^ tumor‐bearing young and old mice and were processed for Targeted TCA analysis. (A–J) Targeted analysis of the TCA cycle metabolites was performed to assess the levels of various metabolites in pooled Tregs from syngeneic mice (*n* = 3) in triplicates. (K) Bar graph represents SDH levels in Tregs isolated from young and old EOC mice. Normalized mRNA expression of (L) SDHa, (M) SDHb, (N) SDHc in Tregs of young and old EOC mice. (O) Immunoblot representing SUCNR1 expression in Tregs of young and old control and EOC mice. (P) Bar graph represents succinate levels in tumor cells of young and old EOC mice. (Q) Bar graph represents succinate levels in ascites of young and old EOC mice. (R) Bar graph represents levels of all TCA metabolites in young and old health and ovarian patients' serum. (S) Bar plot represents individual values of succinate in young and old healthy and ovarian patients' serum from R. *p < 0.05, **p < 0.01, ***p < 0.001 tumor compared to control as assessed by unpaired *T*‐test.

To gain an understanding of the metabolic alterations, we performed a targeted metabolomics in the Tregs by liquid chromatography‐tandem mass spectroscopy (Udumula et al. 2024, 213; Udumula et al. 2023, 23). Analysis of glycolytic metabolites in Tregs revealed no significant differences in glucose (Figure S11A), fructose (Figure S11B), galactitol (Figure S11C), or glucose‐6‐phosphate (Figure S11E) between young and old control or EOC‐bearing mice. However, glycerate levels were elevated in Tregs from old EOC mice compared to their young counterparts (Figure S11G). Additionally, phosphoenolpyruvate (Figure S11D) and mannitol (Figure S11F) levels were increased in Tregs from old EOC mice compared to both young EOC mice and old controls. A striking observation was a strong increase in succinate in Tregs from old mice with EOC compared to young EOC mice, as well as in old non‐EOC mice compared to young non‐EOC mice (Figure 5D). α‐ketoglutarate (αKG) was significantly increased in old control Tregs while it decreased in EOC conditions (Figure 5C), which may indicate a rapid conversion of αKG into succinate. Other downstream metabolites such as fumarate, malate, isocitric acid, and glutamate (Figure 5B,E,F,H) were significantly decreased in old Tregs compared to young Tregs from EOC mice. Citric acid levels decreased in old tumor Tregs when compared to old control Tregs; however, there was no significant difference between young and old tumor Tregs (Figure 5A).

Lactate levels were also significantly decreased in older Tregs when compared to other groups (Figure 5I). Pyruvic acid levels were not changed between any of the groups (Figure 5J). To investigate the mechanism underlying increased succinate levels in aged Tregs, we first measured succinate dehydrogenase (SDH) enzymatic activity using a colorimetric assay. Tregs isolated from older EOC‐bearing mice exhibited significantly reduced SDH activity compared with Tregs from young tumor‐bearing mice (Figure 5K). This decrease in enzymatic activity suggests impaired conversion of succinate to fumarate, consistent with the observed accumulation of succinate in aged Tregs. To determine whether this functional reduction was associated with altered gene expression, we next assessed mRNA levels of individual SDH subunits. Quantitative PCR analysis revealed that transcripts encoding SDH subunits Sdha, Sdhb, and Sdhc were all significantly decreased in Tregs from older mice relative to those from younger mice (Figure 5L–N). Together, these data demonstrate that both SDH enzymatic function and SDH gene expression are diminished in aged Tregs, providing a mechanistic basis for succinate accumulation in the aged tumor microenvironment.

The succinate receptor SUCNR1 (also known as GPR91) was also significantly increased in older Tregs when compared to younger EOC Tregs and healthy controls (Figure 5O). Cancer cells release multiple soluble factors that not only stimulate their growth and metastasis but also regulate immune cells in the TME to facilitate their progression (Cheng et al. 2023, 128; Cortellino and Longo 2023, 2023, 127; Mortazavi Farsani and Verma 2023, 129), an interplay that is crucial for tumor initiation and progression. To investigate whether EOC in older mice contributes to increased succinate levels, we performed colorimetric succinate measurement, which showed an increase in succinate in ascites and tumor cells (Figure 5P,Q). As further validation, targeted metabolomics in serum of aged (≥ 65 years) and younger (≤ 55 years) EOC patients and matched healthy controls corroborated that aged EOC patients exhibited significantly increased succinate levels compared to both younger EOC patients and healthy young and old controls (Figure 5R,S), validating increased succinate being produced by the tumor or TME. This data demonstrates that along with Tregs, the aged TME and tumors have increased succinate levels, which may be taken up by the Tregs.

### 3.7 Tumor Derived Succinate Promotes Treg Function in Aged EOC Mice

To investigate the role of succinate in regulating Treg function, splenic Tregs isolated from young EOC mice were treated with varying concentrations of succinate for 48 h (100 μM, 1, and 5 mM). Succinate resulted in a significant increase in the expression of CD25+ FOXP3 and other immunosuppressive markers, IL‐10 and TGFβ (Figure S12A–C). The addition of succinate to the co‐culture of CD4^+^ T cells and Tregs reduced the proliferation of CD4^+^ T cells (Figure S12D), confirming succinate's crucial role in modulating Treg function. To explore whether succinate from the TME can enhance Treg activity, Tregs from naive mice were exposed to serum from both young and old patients, where serum from old patients led to an increase in FOXP3 expression and immunosuppressive markers IL‐10 and TGFβ (Figure S12E–G), demonstrating that systemic succinate enhances Tregs function. To investigate the impact of tumor‐derived succinate on Tregs, succinate synthesis in tumor cells derived from aged EOC mice was inhibited using CPI‐613, a compound that blocks α‐ketoglutarate dehydrogenase complex (αKGDC), mediated succinate production from αKG (Gao et al. 2020, 109; Khan et al. 2023, 41; Udumula et al. 2024, 213). CPI‐613 led to a reduction in succinate levels in tumor cells compared to untreated cells (Figure S9H). The tumor‐conditioned media (TCM) collected from CPI‐613 treated tumor cells (TCM+ CPI‐613) was then added to Treg cultures, which resulted in a reduction in Tregs, decreased expression of FOXP3, IL‐10 and TGFβ (Figure S12H–K). In addition, the low succinate TCM reversed CD4^+^ T cell suppression compared to TCM from untreated tumor cells (Figure S12L). Moreover, key senescence markers of CD28 was increased and KLRG1 was significantly reduced in the Treg‐CD4 co‐culture exposed to TCM+ CPI‐613, while no significant changes were observed in CD57 and β‐galactosidase (Figure S12M–P). CD8^+^ T cells cultured with TCM exhibited reduced CD28 expression, a co‐stimulatory molecule critical for effective T‐cell activation, along with increased expression of senescence‐associated markers including CD57, KLRG1, and β‐galactosidase. In contrast, exposure to TCM derived from CPI‐613–treated tumor cells (TCM+ CPI‐613) further reduced CD28 expression but significantly decreased the levels of KLRG1, CD57, and β‐galactosidase compared with TCM alone (Figure S12Q–T). These findings suggest that tumor‐derived succinate contributes to the induction of T‐cell senescence, and that reducing succinate levels in the tumor microenvironment can partially alleviate senescence‐associated phenotypes and improve T‐cell functional status. Further, Tregs exposed to TCM+ CPI‐613 displayed decreased OXPHOS when compared to TCM exposed Tregs (Figure S12U,V), while we did not observe any differences in ECAR between TCM and TCM+ CPI‐163 exposed Tregs (Figure S12V–Y). These findings collectively support the conclusion that succinate derived from tumor cells or the TME plays a significant role in enhancing Treg function.

### 3.8 αKGDC Inhibition Improves Overall Survival in Old EOC Mice

**FIGURE 6:**
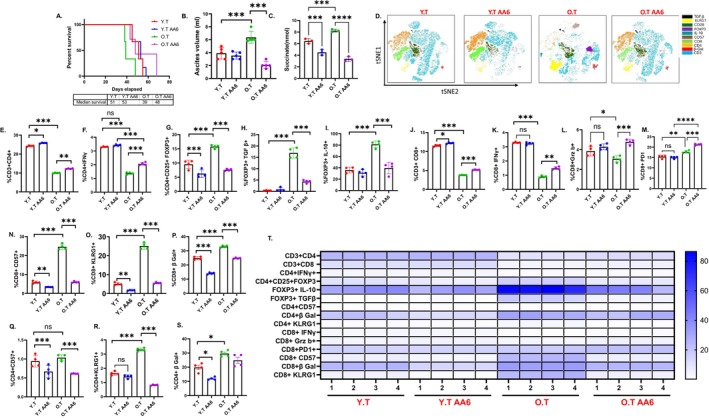
aKGDC inhibition improves overall survival in old EOC mice: Kaplan–Meier graphs indicating overall survival in mice bearing (A) ID8p53−/− cells were injected in mice and were randomly assigned for YT, YT AA6, OT, OT AA6 groups. (B) Bar graphs represent average ascites accumulated in YT, YT AA6, OT, OT AA6 mice. (C) Bar graphs represent succinate levels in ascites of YT, YT AA6, OT, OT AA6 mice. (D) A representative t‐SNE visualization of markers after gating on single, live, CD45, CD4, CD8, IFNγ, FOXP3, IL‐10, TGFβ, CD28, KLRG1, β‐Gal. (E) CD4+, (F) CD4+ IFNγ+, (G) FOXP3, (H) FOXP3+ TGFβ, (I) FOXP3+ IL‐10, (J) CD8+, (K) CD8+ IFNγ+, (L) CD8+ GrzB+, (M) CD8+ PD1, (N) CD8+ CD57, (O) CD8+ KLRG1, (P) CD8+ βGal, (Q) CD4+ CD57, (R) CD4+ KLRG1, (S) CD4+ βGal. (T) Heatmap represents marker expression of the main T cell subsets in individual samples. Immune profiling was performed in 4 individual mice per group. *p < 0.05, **p < 0.01, ***p < 0.001, by one‐way ANOVA, followed by Sidak multiple comparison test.

AA6 is a selective inhibitor of the αKGDC, which blocks the conversion of αKG to succinate (Atlante et al. 2018, 37). We evaluated the effect of AA6 on Tregs; the TCM collected from AA6 treated tumor cells (TCM+ AA6) was then added to Treg cultures, which resulted in a reduction in Tregs, decreased expression of FOXP3, IL‐10, TGFβ, and PD1 (Figure S13A–H). Further we administered AA6 to ID8^p53−/−^ EOC‐bearing young and old mice. AA6 treatment significantly prolonged survival in old EOC mice compared to untreated mice, while no survival benefit was observed in young EOC mice (Figure 6A). Correspondingly, tumor burden, assessed by ascites volume, was significantly reduced in older AA6 treated mice (Figure 6B), while no difference was detected in the young mice. Treatment with AA6 led to a reduction in succinate levels within the ascitic fluid of both young and old mice with ID8^p53−/−^ tumors (Figure 6C). Thus, this could be attributed to low succinate levels in young EOC mice, suggesting a limited therapeutic window for AA6 in this group. Immune profiling revealed an increase in the percentage of CD4^+^ and CD8^+^ T (Figure 6D,E,J) cells in both young and old AA6 treated mice relative to their respective controls. However, enhanced effector function, as observed by increased levels of CD4+ IFNγ+, CD8+ IFNγ+, and CD8+ granzyme B+ (Figure 6F,K,L) T cells, was observed only in aged AA6‐treated mice, while these were unchanged in the young EOC mice. AA6 treatment in EOC old mice increased CD8+ PD1+ percentage in old EOC mice, while it was unchanged in young EOC mice (Figure 6M). More importantly, the frequency of Tregs (Figure 6G) was reduced in both young and old AA6‐treated EOC mice compared to their respective untreated controls. In aged mice, AA6 treatment resulted in decreased immunosuppressive Treg subsets expressing IL10 and TGFβ (Figure 6H,I), an effect not observed in the young group. AA6 treatment also led to a decrease in markers of T cell senescence like CD57, KLRG1, and β‐galactosidase (Figure 6N–P) on CD8^+^ T in both young and old AA6‐treated mice compared to untreated controls. A similar decline was observed in CD4^+^ T (Figure 6Q–S) cells expressing these senescence markers across both age groups. Taken together, these findings demonstrate that AA6 exerts age‐dependent antitumor effects by modulating metabolic pathways, reducing immunosuppression, and attenuating T cell senescence. The therapeutic benefit observed predominantly in aged mice underscores the importance of considering host age and metabolic context when developing targeted therapies for EOC.

## 4 Discussion

Aging is a primary risk factor for developing EOC and other cancers (Foster et al. 2011, 68; Harman 1991, 360; Sedrak and Cohen 2023, 361; Sitnikova et al. 2023, 362). While the average age of EOC diagnosis is 63 years, nearly 45% of women are over 64, and 25% are above 74 years when diagnosed. Unfortunately, EOC outcomes also worsen with age and have poor prognoses (Hightower et al. 1994, 280; Thigpen et al. 1993, 279; Tortorella et al. 2017, 161). Despite this factual relationship between age and EOC, there is limited knowledge about underlying mechanisms. In our study, we demonstrate for the first time, the impaired antitumor T‐cell responses in aged preclinical EOC models, driven by overabundance of immunosuppressive Tregs considering progressive decline of the adaptive immune system is an established hallmark of aging, a better understanding of the intersection between immune aging and tumor progression has widespread implications for human health (Hakim et al. 2004, 228).

Studies have shown that highly immunogenic mouse tumors, such as colon (MC38) and breast (EO771) grow more rapidly in aged animals, while less immunogenic tumors, such as melanoma (B16), do not (Georgiev et al. 2024, 294). EOC is considered a less immunogenic and cold tumor (Blanc‐Durand et al. 2023, 334; Ghisoni et al. 2019, 333), but reports have demonstrated EOC to progress aggressively with increased metastasis in aged mice (Chan et al. 2006, 336; Hou et al. 2022, 11). Our study corroborates these reports and shows decreased survival and increased tumor burden in the ID8 set of EOC cell lines. Aging is associated with a gradual and sustained rise in systemic pro‐inflammatory stress (Isola et al. 2024, 289), often referred to as inflammaging (Di Giosia et al. 2022, 290; Franceschi et al. 2000, 291), and the exhibition of a SASP phenotype (Coleman et al. 2013, 371). We found that in EOC‐bearing old mice the tumor environment represented by ascites had higher levels of SASP cytokines, indicating a close interaction of the EOC and the aged host environment, which encourages tumor growth. In our preclinical models of EOC we demonstrate that aged mice had decreased survival with increased tumor burden when compared to younger mice afflicted with EOC. Interestingly, the modulation of these factors and cytokines differed between the cell line models, indicating tumor phenotype specificity. For example, IFNγ was increased in the ascites of old mice with ID8^p53−/−,BRCA1−/−^ tumors, but decreased in mice with ID8^p53−/−^ tumors, which is due to a more immunosuppressive tumor microenvironment in aged hosts lacking the BRCA1 mutation.

Although age‐associated inflammatory environment and immune decline is well documented (Falvo et al. 2025, 384; Garg et al. 2014, 70), limited studies have investigated the role of an aged immune system in EOC. A study reported that an age‐related increase in metastatic tumor burden was associated with alterations in tumor‐infiltrating lymphocytes and B cell‐related pathways within the gonadal adipose of ovarian tumors (Loughran et al. 2018, 243). However, no studies have profiled the differential immune response in young and old EOC. Studies detailing the change in age‐related distribution and functionality of T cell subsets shift have demonstrated an increase in Tregs with higher immunosuppressive ability in older individuals as a major characteristic (Chen et al. 2024, 296; Elyahu et al. 2019, 343; Garg et al. 2014, 70; Han, Peng, et al. 2023; Han, Georgiev, et al. 2023, 297; Zhao et al. 2023, 298). While the role of Tregs has been demonstrated (Erfani et al. 2014, 340; Ohue and Nishikawa 2019, 338; Zhang et al. 2015, 264) in EOC, it is less explored in aging. Our study shows that old EOC mice have a weaker immune response, marked by reduced antitumor CD4^+^ and CD8^+^ T cells and increased immunosuppressive Tregs with enhanced suppressive activity, compared to young EOC mice. These observations are similar to studies (Georgiev et al. 2024, 299) in aging models of melanoma and colon cancer, which demonstrate a role for CD8^+^ T cells and a potential role of Tregs. This correlation suggests a role for Tregs in suppression of antitumor T cell response and weakening of the adaptive immune response in old mice with EOC. This was supported by the observations that Tregs from old mice with EOC expressed increased immunosuppressive cytokines such as TGFβ and IL‐10.

Furthermore, Tregs isolated from aged EOC‐bearing mice exhibited markedly enhanced immunosuppressive activity, suppressing proliferation and IFNγ production in both CD4^+^ and CD8^+^ T cells. Consistent with these findings, in vivo Treg depletion significantly improved survival and reduced tumor burden, with a more pronounced benefit observed in aged mice. This therapeutic response was associated with increased infiltration of CD4^+^ and CD8^+^ T cells and restoration of effector function, evidenced by elevated CD4^+^IFNγ^+^ and CD8^+^IFNγ^+^ populations and a modest increase in granzyme B expression. Notably, Treg depletion selectively modulated aging‐associated dysfunction by reducing β‐galactosidase and PD‐1 expression while partially restoring CD28 expression, suggesting reversal of key senescence and exhaustion features rather than global immune reprogramming. To distinguish whether enhanced suppression resulted from intrinsic Treg aging or responder T‐cell susceptibility, cross‐over co‐culture experiments were performed using young and aged Tregs with CD8^+^ T cells from both age groups. Aged Tregs retained superior suppressive capacity independent of responder cell age, demonstrating that aging intrinsically enhances Treg function. However, maximal suppression occurred when aged Tregs interacted with aged CD8^+^ T cells, indicating that effector T‐cell aging further increases vulnerability to regulation within the tumor microenvironment. Together, these findings support that aging creates a metabolically altered tumor microenvironment that promotes Treg‐mediated immunosuppression in EOC. In this context, the aged TME appears enriched in metabolites such as succinate, which may enhance Treg metabolic fitness and drive reliance on oxidative phosphorylation, leading to increased expression of suppressive mediators including FOXP3, IL‐10, and TGFβ. This, in turn, is associated with impaired effector T‐cell function and features of senescence, including altered CD28 expression, increased CD57 and KLRG1, and accumulation of DNA damage markers such as γ‐H2AX. However, the precise mechanisms linking age‐associated metabolic changes to Treg function and effector T‐cell dysfunction remain incompletely defined. Ongoing and future studies will be required to dissect these pathways and determine how metabolic rewiring in the aged tumor microenvironment drives immune suppression and tumor progression.

Given the profound age‐associated immune dysfunction, we further examined whether genomic instability contributes to impaired T‐cell responses. CD4^+^ and CD8^+^ T cells from aged tumors accumulated increased DNA damage, which correlated with reduced proliferative capacity and functional decline. DNA damage in aged CD4^+^ T cells may promote stabilization of highly suppressive Treg states, whereas accumulation of damage in CD8^+^ T cells likely limits effective antitumor immunity. Collectively, these findings support a model in which aging‐driven genomic stress and heightened Treg‐mediated suppression cooperatively drive immunosenescence in ovarian cancer. In line with prior studies demonstrating improved responses to immunotherapy following Treg depletion, our data highlight targeting age‐associated Treg dysfunction as a promising strategy to restore antitumor immunity in aged ovarian cancer hosts.

Our results suggest that Treg depletion may have a selective impact, affecting only certain effector‐related and senescence‐related markers. This finding contrasts with other studies, which demonstrate Tregs to suppress T cells by inducing responder T‐cell senescence (Liu et al. 2018, 351; Ye et al. 2012, 249). These findings are consistent with other published studies, which have shown that Treg depletion enhances the effectiveness of immune checkpoint therapies in reducing tumors in melanoma and other cancers (Curtin et al. 2008, 301).

Aging is also associated with systemic metabolic alterations. Aged C57BL/6 mice frequently develop diabetes‐like metabolic dysregulation, including altered insulin and adipokine signaling, which may influence systemic endocrine environments and potentially contribute to metabolic differences observed between young and aged EOC mice (Senkus et al. 2022, 435). Metabolites play a crucial role in the proper functioning of immune and cancer cells. Tregs engage nutrient sensing mechanisms to adapt to both intrinsic and extrinsic environmental cues, triggering metabolic reprogramming to sustain their activity (Kempkes et al. 2019, 173). Tregs have been demonstrated to rely predominantly on OXPHOS and FAO to support their suppressive functions (Field et al. 2020, 400; Zhao 2024, 382), although glycolysis has also been shown to be crucial for their migratory bioenergetic demands. Recent non‐cancer studies have shown dependence of aging Tregs on deactivating oxidative stress while maintaining high OXPHOS and enabling them to function, grow, and activate in older mice (Danileviciute et al. 2022, 304; Guo et al. 2020, 303). Research on aging‐related metabolic changes in Tregs in EOC is limited. Our study is the first to show that Tregs from old EOC mice have higher OXPHOS and ECAR levels than those from young or non‐EOC mice, suggesting greater metabolic fitness with age and cancer. SCENITH analysis showed these Tregs rely on mitochondria, not glycolysis or fatty acid oxidation. Blocking OXPHOS reduced FOXP3 and TGFβ, but not IL‐10, and restored CD4^+^ T cell function. In contrast, blocking glycolysis had no effect on Tregs but impaired CD4^+^ T cells, showing their dependence on glycolysis for activity (Chang et al. 2013, 386; Liu, Zhang, et al. 2023; Liu, Liao, et al. 2023, 385).

The plasticity of Treg metabolism and their ability to utilize tumor/TME metabolites to regulate behavior and function has been well‐documented in multiple cancers (Gu et al. 2022, 286; Moon et al. 2015, 285; Phan and Goldrath 2015, 284; Shi et al. 2011, 287; Wang et al. 2017, 288). However, no age‐specific metabolite in regulation of Tregs in EOC, especially in aged EOC, has been defined. Targeted metabolomics showed that succinate levels in Tregs were higher in old control mice than in young control mice, and even higher in old EOC mice compared to young EOC mice. Succinate, a key metabolite in the TCA cycle, has been implicated in the regulation of immune cell function, including Tregs (Kinsella et al. 2023, 368). SDH enzyme, which converts succinate to fumarate, levels were low in older Tregs when compared to younger Tregs. Further, succinate receptor SUCNR1 was also increased in Tregs isolated from old EOC mice. This suggests that Tregs TCA cycle to promote succinate accumulation as well as ability to imbibe extracellular succinate. Although the impact of succinate on Tregs in cancer has not been explored much, studies have shown that succinate uptake in CD4^+^ T cells suppresses their effector function by inhibiting mitochondrial glucose oxidation (Gudgeon et al. 2022, 281). A recent study elegantly showed extracellular succinate to promote Treg numbers in lung cancer and melanoma cells (Kinsella et al. 2023, 368). Other research has demonstrated that extracellular succinate can polarize macrophages into the M2 phenotype through SUCNR1 signaling (Harber et al. 2020, 283; Trauelsen et al. 2021, 282). In our studies, Tregs isolated from younger mice exposed to succinate resulted in a dose‐dependent increase in the expression of FOXP3, TGFβ, and IL‐10, thereby enhancing their immunosuppressive phenotype, as functionally seen by suppression of CD4^+^ T cell proliferation in a dose‐dependent manner. Upregulated SUCNR1 in Tregs from old mice with EOC indicated increased potential to uptake succinate, which raised the questions if EOC cells and TME are sources of succinate. We found that tumors and ascites from old EOC mice did indeed have elevated succinate. This was further confirmed in patients where serum from EOC patients over the age of 65 demonstrated significantly increased succinate compared to other TCA cycle metabolites, in comparison to EOC patients below 55 years of age. Exposure of the patient serum or ascites from old EOC mice to Tregs from naïve young mice increased the Treg number as well as the expression of immunosuppressive markers. As further proof, when succinate production in tumor cells derived from aged mice was inhibited, the conditioned media exposure with low succinate resulted in a marked reduction of FOXP3 expression and immunosuppressive cytokines TGFβ and IL‐10, indicating a decrease in the suppressive phenotype of Tregs, confirmed by reversal of CD4^+^ T cell proliferation. Succinate inhibition also led to a reversal in the expression of key senescence markers, including CD28 and KLRG1. These results indicate a pivotal role of succinate in regulating immunosuppressive Treg function in an aged TME of EOC. To the relevance of succinate in vivo, we employed a relatively more specific αKGDC inhibitor, AA6. AA6 treatment improved survival and reduced tumor burden in old EOC mice but had no effect on young mice. It decreased FOXP3^+^ cells in both groups, while reducing immunosuppressive IL‐10 and TGFβ only in old mice. In old mice, AA6 also restored antitumor immunity by increasing CD4^+^, CD8^+^, and cytotoxic T cells and lowering senescence markers. Inhibition of αKGDC also resulted in increased PD‐1 expression on CD8^+^ T cells, which may reflect enhanced T cell activation (Simon and Labarriere 2017, 367). Collectively, our study demonstrates that the aged TME of EOC is characterized by elevated succinate, which is key in promoting Treg‐mediated immunosuppression probably by ensuring metabolic fitness and contributing to effective immune escape in elderly EOC patients. Targeting succinate metabolism may therefore offer a promising strategy for modulating the immune landscape and restoring antitumor immunity in aging individuals.

However, our study has several limitations. Inhibition of the α‐ketoglutarate dehydrogenase complex (αKGDC) by CPI‐613 or AA6 affects multiple steps of the TCA cycle rather than selectively reducing succinate production. Blocking αKGDC can lead to the accumulation of α‐ketoglutarate (αKG) in addition to altering downstream succinate levels, meaning that both metabolites may contribute to the observed effects. αKG itself is an active metabolite known to regulate signaling and epigenetic processes. Previous studies have shown that αKG can have direct antitumor effects, regulate cytotoxic T‐cell function and proliferation, and serve as an important cofactor for TET enzymes involved in DNA demethylation (Tran et al. 2019, 442; Liu, Zhang, et al. 2023; Liu, Liao, et al. 2023, 443). In addition, αKG has been reported to have anti‐aging effects by modulating cellular senescence (Rhoads and Anderson 2020, 440; Sandalova et al. 2023, 441). Therefore, the immune and tumor phenotypes observed after αKGDC inhibition may reflect the combined effects of changes in both succinate and αKG levels. Our current study does not directly separate the specific contribution of αKG accumulation from the effects of reduced succinate in EOC.

Our findings suggest that succinate may act as an important metabolic regulator of Treg function in the aged ovarian tumor microenvironment. However, the precise source of elevated succinate within Tregs remains unresolved. Succinate could arise from intrinsic metabolic rewiring of the TCA cycle within Tregs or from uptake of extracellular succinate present in the tumor or ascites microenvironment through SUCNR1 signaling. In support of the latter possibility, exposure to tumor‐conditioned media, ascites, or patient serum enhanced Treg‐associated suppressive phenotypes, while pharmacological inhibition of SUCNR1 reduced FOXP3 expression and the production of suppressive cytokines such as IL‐10 and TGFβ. These observations suggest that tumor‐derived metabolites may reinforce Treg‐mediated immunosuppression in aged hosts. Future studies will be required to dissect the relative contribution of intrinsic metabolic remodeling versus extracellular succinate signaling in shaping Treg function during aging‐associated ovarian cancer progression.

Most of the experiments in this study were performed using the ID8p53^−^/^−^ EOC model, which is clinically relevant because TP53 mutations occur in more than 90% of HGSOC patients (Garziera et al. 2018, 445). Our analysis with ID8 p53−/−, BRCA1−/− displayed similar immune and metabolic changes with aging, including altered T cell responses and increased immunosuppressive pathways. Based on these consistent findings, we focused our mechanistic studies on this representative model, while future studies using additional EOC models will further validate the broader applicability of our results.

Despite this, our study implies a crucial role for Tregs in driving aggressive EOC due to increased immunosuppression in an already age‐compromised environment. Thus, understanding the changes and mechanisms of Treg dynamics with age can help tailor precision‐based therapeutics including immunotherapy for older EOC patients.

## Author Contributions

M.P.U. designed the experiments, conducted research, analyzed the data, wrote and edited the manuscript. A.K.M., M.N., F.R., H.S., and T.B. performed research and analyzed data. M.H., S.G., E.N.C., and H.M.G. analyzed the data and edited manuscript. R.R. designed experiments, supervised the research, analyzed the data and edited the manuscript.

## Funding

This work was supported by NIH/NCI R01CA249188 to R.R. M.P.U. is supported by Henry Ford Health Internal Mentored Award (A20091). K.M. is partially supported by Henry Ford Cancer post‐doctoral fellowship award.

## Conflicts of Interest

The authors declare no conflicts of interest.

## Supporting information


**Figure S1:** Aging exacerbates preclinical EOC models: ID8‐luc2 cells were injected in young and old EOC mice, (A). Representative BLI images at 4 weeks of tumor inoculation, (B) Bar graph of BLI quantification of images in A. ***p < 0.001, OT compared with YT group by Student's *t*‐test.
**Figure S2:** Aging enhances tumor promoting metabolic growth factors and Senescence Associated Secretory Phenotype associated inflammation: ID8p53−/− and ID8 P53−/−, BRCA 1−/− EOC cells were injected in YT or OT (*n* = 10/group), ascites was collected and ELISA was performed for key inflammatory and growth factors. (A–X) Measurement of (A, E) Insulin, (B, F) IGF‐1, (C, G) Adiponectin, (D, H) Leptin, (I, M) IL‐4, (J, N) MCP‐1, (K, O) IL‐10, (L, P) GMCSF, (Q, U) IL‐1β, (R, V) TNFα, (S, W) IL‐6, (T, X) IFN γ, and in ascites collected at week 5 from ID8p53−/− and ID8p53−/−, BRCA 1−/− EOC bearing mice by ELISA (*n* = 3). *p < 0.05, **p < 0.01, ***p < 0.001, OT compared with YT group by Student's *t*‐test.
**Figure S3:** Aging induces differential systemic T cell response in response to EOC: ID8p53−/− EOC injected in Y.C, YT or O.C, OT (*n* = 10/group), blood was collected and processed for immune profiling. (A–I) (A) A representative t‐SNE visualization of markers after gating on single, live, CD45+ CD3+, CD4+, CD8+, IFNγ, Granzyme B (Grz B), perforin and FOXP3 expression. (B) Heatmap represents marker expression of the main T cell subsets in individual samples. Immune profiling was performed in blood of 5 individual mice per group. Bar plots represent the percentage of T cell subsets (C) CD4+, (D) CD4+ IFNγ+, (E) CD4+ CD25+ FOXP3 (F) CD8+, (G) CD8+ IFNγ+, (H) CD8+ Grzb+, (I) CD8+ Perforin+, (J) CD11b+ F4/80, (K) F4/80+ CD38, (L) F4/80+ EGR2, (M) F4/80+ CD206, (N) CD38/EGR2, (O) CD38/CD206, (P) CD38+ iNOS, (Q) EGR2+ Arg1, (R) CD206+ Arg1, (S) CD11b+ GR1. The experiment was repeated twice in two different sets of mouse experiments. *p < 0.05, **p < 0.01, ***p < 0.001.
**Figure S4:** Aging diminishes the T cell response in EOC TME: ID8p53−/− EOC injected in YT or OT (*n* = 10/group), Immune profiling was performed in ascites of 5 individual mice per group (A–R) (A) A representative t‐SNE visualization of markers after gating on single, live, CD45+ CD3+, CD4+, CD8+, IFNγ, Granzyme B, perforin and PD1 expression. (B) Heatmap represents marker expression of the main T cell subsets in individual samples. Bar plots represent the percentage of T cell subsets (C) CD8+, (D) CD8+ IFNγ+, (E) CD8+ GrzB+, (F) CD8+ perforin, (G) CD8+ PD1. (H) A representative t‐SNE visualization of markers after gating on single, live, CD45+ CD3+, CD4+, IFNγ, and Tbet expression. (I) Heatmap represents marker expression of the main T cell subsets in individual samples. Bar plots represent (J) CD4, (K) CD4+ IFNγ+ (L) CD4+ Tbet. (M) A representative t‐SNE visualization of markers after gating on single, live, CD45+ CD3+, CD4+, CD25+, FOXP3, IL‐10, TGF‐β and PD1 expression. (N) Heatmap represents marker expression of the main T cell subsets in individual samples. Bar plots represent the percentage of T cell subsets (O) FOXP3, (P) FOXP3+ TGF‐β, (Q) FOXP3+ IL‐10, (R) FOXP3+ PD1 in the ascites. The experiment was repeated twice in two different sets of mouse experiments. *p < 0.05, **p < 0.01, ***p < 0.001, OT compared to YT group by Student's *t*‐test.
**Figure S5:** Aging diminishes the intra‐tumor T cell response in EOC: ID8p53−/−, BRCA 1−/− EOC injected in YT or OT (*n* = 10/group), Immune profiling was performed in tumors of 5 individual mice per group (A–K) (A) A representative t‐SNE visualization of markers after gating on single, live, CD45+ CD3+, CD4+, CD8+, IFNγ, Granzyme B (Grz B), perforin, Tbet and FOXP3. (B) Bar plots represent the percentage of T cell subsets (B) CD4+ (C) CD4+ IFNγ, (D) CD4+ Tbet, (E) CD25+ FOXP3, (F) FOXP3+ IL‐10, (G) FOXP3+ TGFβ, (H) CD8+, (I) CD8+ IFNγ, (J) CD8+ Granzyme B, (K) CD8+ Perforin. The experiment was repeated twice in two different sets of mouse experiments. **p < 0.01, ***p < 0.001, OT compared to YT group by Student's *t*‐test.
**Figure S6:** Aging diminishes the ascitic T cell response in EOC: ID8p53−/−, BRCA 1−/− EOC injected in YT or OT (*n* = 10/group), Immune profiling was performed in the ascites of 4 individual mice per group (A–K) (A) A representative t‐SNE visualization of markers after gating on single, live, CD45+ CD3+, CD4+, CD8+, IFNγ, Granzyme B (Grz B), perforin, Tbet and FOXP3. (B) Bar plots represent the percentage of T cell subsets (B) CD4+ (C) CD4+ IFNγ, (D) CD4+ Tbet, (E) CD25+ FOXP3, (F) FOXP3+ IL‐10, (G) FOXP3+ TGFβ, (H) CD8+, (I) CD8+ IFNγ, (J) CD8+ Granzyme B, (K) CD8+ Perforin. The experiment was repeated twice in two different sets of mouse experiments. **p < 0.01, ***p < 0.001, OT compared to YT group by Student's *t*‐test.
**Figure S7:** Tregs are key in promoting EOC: ID8p53−/− EOC cells were injected in YT or OT mice (*n* = 10/group), Immune profiling was performed in the tumors of 5–6 individual mice per group. (A) Experimental plan showing the Treg depletion strategy. (B)Kaplan–Meier graph indicating overall survival (*n* = 10), *p* = 0.002 by Gehan‐Breslow‐Wilcoxon test. (C) Average ascites accumulated in OT, O. Treg dep. Immune profiling was performed in blood (D) A representative t‐SNE visualization of markers after gating on single, live, CD45, CD4, CD8, IFNγ, Granzyme B (Grz B), perforin and PD1. Bar plots represent the percentage of senescence markers on T cell subsets (E) CD4+ (F) CD4+ IFNγ+, (G) FOXP3, (H) FOXP3+ IL‐10, (I) FOXP3+ TGFβ, (J) Heatmap represents marker expression of the main T cell subsets in individual samples. (K) CD8+, (L) CD8+ IFNγ+, (M) CD8+ perforin, (N) CD8+ GrzB+, (O) CD8+ PD1, (P) CD8+ KLRG1, (Q) CD8+ βGal, (R) CD8+ CD57, (S) CD8+ CD28 (T) CD4+ KLRG1, (U) CD4+ βGal, (V) CD4+ CD57, (W) CD4+ CD28. Immune profiling was performed in 5 individual mice per group. *p < 0.05, **p < 0.01, ***p < 0.001, by one‐way ANOVA, followed by Sidak multiple comparison test.
**Figure S8:** Tregs are key in promoting EOC: ID8p53−/− EOC cells were injected in YT or OT mice (*n* = 10/group), Immune profiling was performed in the ascites of 4–5 individual mice per group. (A) A representative t‐SNE visualization of markers after gating on single, live, CD45, CD4, CD8, IFNγ, Granzyme B (Grz B), perforin and PD1. Bar plots represent the percentage of senescence markers on T cell subsets (B) CD4+, (C) CD4+ IFNγ+, (D) FOXP3, (E) FOXP3+ IL‐10, (F) FOXP3+ TGFβ, (G) Heatmap represents marker expression of the main T cell subsets in individual samples. (H) CD8+, (I) CD8+ IFNγ+, (J) CD8+ perforin, (K) CD8+ GrzB+, (L) CD8+ PD1, (M) CD8+ KLRG1, (N) CD8+ βGal, (O) CD8+ CD57, (P) CD8+ CD28, (Q) CD4+ KLRG1, (R) CD4+ βGal, (S) CD4+ CD57, (T) CD4+ CD28. Immune profiling was performed in 4 individual mice per group. *p < 0.05, **p < 0.01, ***p < 0.001, by one‐way ANOVA, followed by Sidak multiple comparison test.
**Figure S9:** Tregs are key in promoting EOC: ID8p53−/− EOC cells were injected in YT or OT mice (*n* = 10/group), Immune profiling was performed in the blood of 4 individual mice per group. (A) A representative t‐SNE visualization of markers after gating on single, live, CD45, CD4, CD8, IFNγ, Granzyme B (Grz B), perforin and PD1. Bar plots represent the percentage of senescence markers on T cell subsets (B) CD4+, (C) CD4+ IFNγ+, (D) FOXP3, (E) FOXP3+ IL‐10, (F) FOXP3+ TGFβ, (G) Heatmap represents marker expression of the main T cell subsets in individual samples. (H) CD8+, (I) CD8+ IFNγ+, (J) CD8+ perforin, (K) CD8+ GrzB+, (L) CD8+ PD1, (M) CD8+ KLRG1, (N) CD8+ βGal, (O) CD8+ CD57, (P) CD8+ CD28, (Q) CD4+ KLRG1, (R) CD4+ βGal, (S) CD4+ CD57, (T) CD4+ CD28. Immune profiling was performed in 4 individual mice per group. *p < 0.05, **p < 0.01, ***p < 0.001, by one‐way ANOVA, followed by Sidak multiple comparison test.
**Figure S10:** Tregs from aged EOC mice prefer OXPHOS for immunosuppression: ID8p53−/−, BRCA 1−/− EOC cells were injected in YT or OT, FOXP3+ cells were isolated from the spleens of EOC young and old tumor at 5 weeks and subjected to Seahorse analysis and energy targeted metabolomics. (A) Oxygen consumption rate (OCR) was assessed in real‐time using an XFe 96 Seahorse analyzer as described in methods. Port injections of (1) oligomycin, (2) FCCP, and a combination of (3) rotenone‐antimycin were given. (B) The bar graph represents basal and stressed OCR (*n* = 3). (C) Extracellular acidification rate (ECAR) was measured with port injections of (1) glucose, (2) oligomycin, and (3) 2‐DG. (D) The bar graph represents basal and stressed ECAR (*n* = 3). ***p < 0.001, OT compared to YT group by Student's *t*‐test.
**Figure S11:** Altered Glycolytic Metabolite Profiles in Young and Aged Regulatory T Cells: Tregs were isolated from spleens of control and p53−/− tumor bearing young and old mice and were processed for Glycolysis metabolites. (A‐G) Targeted analysis of the major glycolysis metabolites was performed to assess the levels of various metabolites from pooled syngeneic mice (*n* = 3) in triplicates.
**Figure S12:** Tumor derived succinate promotes Treg function in aged EOC mice: Tregs isolated from young p53−/− EOC mice and cultured with and without succinate (Succinate 100 μM, 1, 5 mM). Bar graph shows the percentage of A. CD25+ FOXP3 cells. B. FOXP3+ IL10 and C. FOXP3+ TGFβ. D. CD4+ CFSE from Tregs cocultured with naïve CD4 T cells in the presence and absence of succinate (100 μM, 1, 5 mM). Tregs from young EOC mice were exposed to young and old patient serum and flow analysis was performed Bar graph represents (E) %CD25+ FOXP3, (F) FOXP3+ IL‐10, (G) FOXP3+ TGF‐β. Tumors cells from aged EOC mice were treated with CPI‐613 and the tumor condition media (TCM) and CPI‐613+ TCM media was exposed to Tregs from young EOC mice. Bar graph represents the percentage of (H) succinate levels in the tumor cells after treatment with CPI‐613. (I) CD25+ FOXP3, (J) FOXP3+ IL‐10, (K) FOXP3+ TGFβ. (L) CFSE proliferation histograms and bar graph representing CD4 proliferation in the presence of Tregs exposed to TCM and CPI‐613+ TCM. Flow analysis was performed in the co‐culture experiment, and bar graph represents senescence markers (M) CD4+ CD28, (N) CD4+ KLRG1, (O) CD4+ CD57, (P) CD4+ β‐Gal. (Q) CD8+ CD28, (R) CD8+ KLRG1, (S) CD8+ CD57, (T) CD8+ β‐Gal. Bioenergetics using seahorse was performed on Tregs exposed to TCM and CPI‐613+ TCM (U) Oxygen consumption rate (OCR) was assessed in real‐time using an XFe96 Seahorse analyzer as described in methods. Port injections of (1) oligomycin, (2) FCCP, and a combination of (3) rotenone‐antimycin were given. (V) The bar graph represents basal and stressed OCR (*n* = 3). (W) Extracellular acidification rate (ECAR) was 5 measured with port injections of (1) glucose, (2) oligomycin, and (3) 2‐DG. (X) The bar graph represents basal and stressed ECAR (*n* = 3). (Y) A quadrant plot indicates the metabolic shift of energy phenotype at basal and stressed levels. ***p < 0.001, **p < 0.01, *p < 0.05.
**Figure S13:** Succinate inhibition reduces Treg function: Tumors cells from aged EOC mice were treated with AA6 and the tumor condition media (TCM) and TCM+ AA6 media was exposed to Tregs from young P53−/− EOC mice. Bar graph represents the percentage of (A, E) CD25+ FOXP3, (C, H) FOXP3+ IL‐10, (B, F) FOXP3+ TGFβ and (D, G) PD1. ***p < 0.001, TCM+ AA6 compared with TCM group by Student's *t*‐test.
**Figure S14:** Age‐dependent regulatory T cell–mediated suppression differentially impairs CD8^+^ T‐cell responses: Tregs isolated from young or aged ID8p53−/− EOC tumor‐bearing mice were co‐cultured with CD8^+^ T cells derived from either young or aged naïve conditions. (A–D) Aged Tregs were co‐cultured with young or aged CD8^+^ T cells. Immune profiling was performed for CFSE and key cytotoxic CD8 markers. The bar graph represents the percentage of (A) CD8+ CFSE, (B) CD8+ Gnzm b, (C) CD8+ IFNγ, (D) CD8+ Perforin. (E–H) Young Tregs were co‐cultured with young or aged CD8^+^ T cells. Immune profiling was performed for CFSE and key cytotoxic CD8 markers. The bar graph represents the percentage of (E) CD8+ CFSE, (F) CD8+ Gnzm b, (G) CD8+ IFNγ, (H) CD8+ Perforin. ***p < 0.001, OT compared with the YT group by Student's *t*‐test.

## Data Availability

This study does not analyze any publicly available datasets. All data supporting the findings of this study are available from the corresponding author upon reasonable request. No original code was generated or used in this study. Any additional information necessary to reanalyze the reported data can be obtained from the corresponding author upon request.

## References

[acel70510-bib-0001] Almohaimeed, H. M. , H. Waly , N. S. Abou Khalil , K. M. A. Hassanein , B. S. M. Alkhudhairy , and E. A. Abd‐Allah . 2022. “Gum Arabic Nanoformulation Rescues Neuronal Lesions in Bromobenzene‐Challenged Rats by Its Antioxidant, Anti‐Apoptotic and Cytoprotective Potentials.” Scientific Reports 12: 21213. 10.1038/s41598-022-24556-0. 36481816 PMC9731957

[acel70510-bib-0002] Anisimov, V. N. 2007. “Biology of Aging and Cancer.” Cancer Control 14: 23–31. 10.1177/107327480701400104. 17242668

[acel70510-bib-0003] Arguello, R. J. , A. J. Combes , R. Char , et al. 2020. “SCENITH: A Flow Cytometry‐Based Method to Functionally Profile Energy Metabolism With Single‐Cell Resolution.” Cell Metabolism 32: 1063–1075. 10.1016/j.cmet.2020.11.007. 33264598 PMC8407169

[acel70510-bib-0005] Atlante, S. , A. Visintin , E. Marini , et al. 2018. “Alpha‐Ketoglutarate Dehydrogenase Inhibition Counteracts Breast Cancer‐Associated Lung Metastasis.” Cell Death & Disease 9: 756. 10.1038/s41419-018-0802-8. 29988033 PMC6037705

[acel70510-bib-0006] Blanc‐Durand, F. , L. Clemence Wei Xian , and D. S. P. Tan . 2023. “Targeting the Immune Microenvironment for Ovarian Cancer Therapy.” Frontiers in Immunology 14: 1328651. 10.3389/fimmu.2023.1328651. 38164130 PMC10757966

[acel70510-bib-0007] Bottazzi, B. , E. Riboli , and A. Mantovani . 2018. “Aging, Inflammation and Cancer.” Seminars in Immunology 40: 74–82. 10.1016/j.smim.2018.10.011. 30409538

[acel70510-bib-0008] Caruso, C. , D. Lio , L. Cavallone , and C. Franceschi . 2004. “Aging, Longevity, Inflammation, and Cancer.” Annals of the New York Academy of Sciences 1028: 1–13. 10.1196/annals.1322.001. 15915584

[acel70510-bib-0009] Cassar, E. , A. E. R. Kartikasari , and M. Plebanski . 2022. “Regulatory T Cells in Ovarian Carcinogenesis and Future Therapeutic Opportunities.” Cancers 14: 5488. 10.3390/cancers14225488. 36428581 PMC9688690

[acel70510-bib-0010] Chan, J. K. , R. Urban , M. K. Cheung , et al. 2006. “Ovarian Cancer in Younger vs Older Women: A Population‐Based Analysis.” British Journal of Cancer 95: 1314–1320. 10.1038/sj.bjc.6603457. 17088903 PMC2360593

[acel70510-bib-0011] Chang, C. H. , J. D. Curtis , L. B. Maggi Jr. , et al. 2013. “Posttranscriptional Control of T Cell Effector Function by Aerobic Glycolysis.” Cell 153: 1239–1251. 10.1016/j.cell.2013.05.016. 23746840 PMC3804311

[acel70510-bib-0012] Chapman, J. R. , A. J. Sossick , S. J. Boulton , and S. P. Jackson . 2012. “BRCA1‐Associated Exclusion of 53BP1 From DNA Damage Sites Underlies Temporal Control of DNA Repair.” Journal of Cell Science 125: 3529–3534. 10.1242/jcs.105353. 22553214 PMC3445322

[acel70510-bib-0013] Chavez, M. D. , and H. M. Tse . 2021. “Targeting Mitochondrial‐Derived Reactive Oxygen Species in T Cell‐Mediated Autoimmune Diseases.” Frontiers in Immunology 12: 703972. 10.3389/fimmu.2021.703972. 34276700 PMC8281042

[acel70510-bib-0014] Chen, A. C. Y. , S. Jaiswal , D. Martinez , et al. 2024. “The Aged Tumor Microenvironment Limits T Cell Control of Cancer.” Nature Immunology 25: 1033–1045. 10.1038/s41590-024-01828-7. 38745085 PMC11500459

[acel70510-bib-0015] Chen, M. L. , M. J. Pittet , L. Gorelik , et al. 2005. “Regulatory T Cells Suppress Tumor‐Specific CD8 T Cell Cytotoxicity Through TGF‐Beta Signals In Vivo.” Proceedings of the National Academy of Sciences of the United States of America 102: 419–424. 10.1073/pnas.0408197102. 15623559 PMC544311

[acel70510-bib-0016] Cheng, J. , J. Yan , Y. Liu , et al. 2023. “Cancer‐Cell‐Derived Fumarate Suppresses the Anti‐Tumor Capacity of CD8(+) T Cells in the Tumor Microenvironment.” Cell Metabolism 35: 961–978. 10.1016/j.cmet.2023.04.017. 37178684

[acel70510-bib-0017] Churov, A. V. , K. Y. Mamashov , and A. V. Novitskaia . 2020. “Homeostasis and the Functional Roles of CD4(+) Treg Cells in Aging.” Immunology Letters 226: 83–89. 10.1016/j.imlet.2020.07.004. 32717201

[acel70510-bib-0018] Coleman, P. R. , G. Chang , G. Hutas , M. Grimshaw , M. A. Vadas , and J. R. Gamble . 2013. “Age‐ Associated Stresses Induce an Anti‐Inflammatory Senescent Phenotype in Endothelial Cells.” Aging 5: 913–924. 10.18632/aging.100622. 24334613 PMC3883707

[acel70510-bib-0019] Cortellino, S. , and V. D. Longo . 2023. “Metabolites and Immune Response in Tumor Microenvironments.” Cancers 15: 3898. 10.3390/cancers15153898. 37568713 PMC10417674

[acel70510-bib-0020] Cura Daball, P. , M. S. Ventura Ferreira , S. Ammann , et al. 2018. “CD57 Identifies T Cells With Functional Senescence Before Terminal Differentiation and Relative Telomere Shortening in Patients With Activated PI3 Kinase Delta Syndrome.” Immunology and Cell Biology 96: 1060–1071. 10.1111/imcb.12169. 29790605

[acel70510-bib-0021] Curtin, J. F. , M. Candolfi , T. M. Fakhouri , et al. 2008. “Treg Depletion Inhibits Efficacy of Cancer Immunotherapy: Implications for Clinical Trials.” PLoS One 3: e1983. 10.1371/journal.pone.0001983. 18431473 PMC2291560

[acel70510-bib-0022] Danileviciute, E. , N. Zeng , C. M. Capelle , et al. 2022. “PARK7/DJ‐1 Promotes Pyruvate Dehydrogenase Activity and Maintains T(Reg) Homeostasis During Ageing.” Nature Metabolism 4: 589–607. 10.1038/s42255-022-00576-y. 35618940

[acel70510-bib-0023] Deng, F. , X. Xu , M. Lv , et al. 2017. “Age Is Associated With Prognosis in Serous Ovarian Carcinoma.” Journal of Ovarian Research 10: 36. 10.1186/s13048-017-0331-6. 28606125 PMC5469143

[acel70510-bib-0024] Di Giosia, P. , C. A. Stamerra , P. Giorgini , T. Jamialahamdi , A. E. Butler , and A. Sahebkar . 2022. “The Role of Nutrition in Inflammaging.” Ageing Research Reviews 77: 101596. 10.1016/j.arr.2022.101596. 35219904

[acel70510-bib-0025] Dong, Z. , Y. Luo , Z. Yuan , Y. Tian , T. Jin , and F. Xu . 2024. “Cellular Senescence and SASP in Tumor Progression and Therapeutic Opportunities.” Molecular Cancer 23: 181. 10.1186/s12943-024-02096-7. 39217404 PMC11365203

[acel70510-bib-0026] Dowling, M. R. , A. Kan , S. Heinzel , J. M. Marchingo , P. D. Hodgkin , and E. D. Hawkins . 2018. “Regulatory T Cells Suppress Effector T Cell Proliferation by Limiting Division Destiny.” Frontiers in Immunology 9: 2461. 10.3389/fimmu.2018.02461. 30425712 PMC6218578

[acel70510-bib-0027] Dutta, S. , and P. Sengupta . 2016. “Men and Mice: Relating Their Ages.” Life Sciences 152: 244–248. 10.1016/j.lfs.2015.10.025. 26596563

[acel70510-bib-0028] Effros, R. B. 1997. “Loss of CD28 Expression on T Lymphocytes: A Marker of Replicative Senescence.” Developmental and Comparative Immunology 21: 471–478. 10.1016/s0145-305x(97)00027-x. 9463780

[acel70510-bib-0029] Ehinger, J. K. , S. Piel , R. Ford , et al. 2016. “Cell‐Permeable Succinate Prodrugs Bypass Mitochondrial Complex I Deficiency.” Nature Communications 7: 12317. 10.1038/ncomms12317. PMC4980488 27502960

[acel70510-bib-0030] Elyahu, Y. , I. Hekselman , I. Eizenberg‐Magar , et al. 2019. “Aging Promotes Reorganization of the CD4 T Cell Landscape Toward Extreme Regulatory and Effector Phenotypes.” Science Advances 5: eaaw8330. 10.1126/sciadv.aaw8330. 31457092 PMC6703865

[acel70510-bib-0031] Erfani, N. , M. Hamedi‐Shahraki , S. Rezaeifard , M. Haghshenas , M. Rasouli , and A. Samsami Dehaghani . 2014. “FoxP3+ Regulatory T Cells in Peripheral Blood of Patients With Epithelial Ovarian Cancer.” Iranian Journal of Immunology 11: 105–112. 24975967 10.22034/iji.2014.16771

[acel70510-bib-0032] Ershler, W. B. , and D. L. Longo . 1997. “Aging and Cancer: Issues of Basic and Clinical Science.” Journal of the National Cancer Institute 89: 1489–1497. 10.1093/jnci/89.20.1489. 9337345

[acel70510-bib-0033] Falvo, P. , S. Gruener , S. Orecchioni , et al. 2025. “Age‐Dependent Differences in Breast Tumor Microenvironment: Challenges and Opportunities for Efficacy Studies in Preclinical Models.” Cell Death and Differentiation 32: 1000–1013. 10.1038/s41418-025-01447-1. 39870804 PMC12162869

[acel70510-bib-0034] Fane, M. , and A. T. Weeraratna . 2020. “How the Ageing Microenvironment Influences Tumour Progression.” Nature Reviews. Cancer 20: 89–106. 10.1038/s41568-019-0222-9. 31836838 PMC7377404

[acel70510-bib-0035] Field, C. S. , F. Baixauli , R. L. Kyle , et al. 2020. “Mitochondrial Integrity Regulated by Lipid Metabolism Is a Cell‐Intrinsic Checkpoint for Treg Suppressive Function.” Cell Metabolism 31: 422–437. 10.1016/j.cmet.2019.11.021. 31883840 PMC7001036

[acel70510-bib-0036] Foster, A. D. , A. Sivarapatna , and R. E. Gress . 2011. “The Aging Immune System and Its Relationship With Cancer.” Aging Health 7: 707–718. 10.2217/ahe.11.56. 22121388 PMC3222953

[acel70510-bib-0037] Franceschi, C. , M. Bonafe , S. Valensin , et al. 2000. “Inflamm‐Aging. An Evolutionary Perspective on Immunosenescence.” Annals of the New York Academy of Sciences 908: 244–254. 10.1111/j.1749-6632.2000.tb06651.x. 10911963

[acel70510-bib-0038] Gao, L. , Z. Xu , Z. Huang , et al. 2020. “CPI‐613 Rewires Lipid Metabolism to Enhance Pancreatic Cancer Apoptosis via the AMPK‐ACC Signaling.” Journal of Experimental & Clinical Cancer Research 39: 73. 10.1186/s13046-020-01579-x. 32345326 PMC7187515

[acel70510-bib-0039] Garg, S. K. , C. Delaney , T. Toubai , et al. 2014. “Aging Is Associated With Increased Regulatory T‐Cell Function.” Aging Cell 13: 441–448. 10.1111/acel.12191. 24325345 PMC4032602

[acel70510-bib-0040] Garrido‐Rodriguez, V. , I. Herrero‐Fernandez , M. J. Castro , et al. 2021. “Immunological Features Beyond CD4/CD8 Ratio Values in Older Individuals.” Aging 13: 13443–13459. 10.18632/aging.203109. 34038386 PMC8202849

[acel70510-bib-0141] Garziera, M. , E. Cecchin , V. Canzonieri , et al. 2018. “Identification of Novel Somatic TP53 Mutations in Patients with High‐Grade Serous Ovarian Cancer (HGSOC) Using Next‐Generation Sequencing (NGS).” International Journal of Molecular Sciences 19, no. 5: 1510. 29783665 10.3390/ijms19051510 PMC5983728

[acel70510-bib-0041] Geels, S. N. , A. Moshensky , R. S. Sousa , et al. 2024. “Interruption of the Intratumor CD8(+) T Cell:Treg Crosstalk Improves the Efficacy of PD‐1 Immunotherapy.” Cancer Cell 42: 1051–1066. 10.1016/j.ccell.2024.05.013. 38861924 PMC11285091

[acel70510-bib-0042] Georgiev, P. , S. Han , A. Y. Huang , et al. 2024. “Age‐Associated Contraction of Tumor‐Specific T Cells Impairs Antitumor Immunity.” Cancer Immunology Research 12: 1525–1541. 10.1158/2326-6066.CIR-24-0463. 39186561 PMC11532741

[acel70510-bib-0043] Ghisoni, E. , M. Imbimbo , S. Zimmermann , and G. Valabrega . 2019. “Ovarian Cancer Immunotherapy: Turning Up the Heat.” International Journal of Molecular Sciences 20: 2927. 10.3390/ijms20122927. 31208030 PMC6628106

[acel70510-bib-0044] Gonzalez‐Navajas, J. M. , D. D. Fan , S. Yang , et al. 2021. “The Impact of Tregs on the Anticancer Immunity and the Efficacy of Immune Checkpoint Inhibitor Therapies.” Frontiers in Immunology 12: 625783. 10.3389/fimmu.2021.625783. 33717139 PMC7952426

[acel70510-bib-0045] Goschl, L. , T. Preglej , P. Hamminger , et al. 2018. “A T Cell‐Specific Deletion of HDAC1 Protects Against Experimental Autoimmune Encephalomyelitis.” Journal of Autoimmunity 86: 51–61. 10.1016/j.jaut.2017.09.008. 28964722

[acel70510-bib-0046] Gu, J. , J. Zhou , Q. Chen , et al. 2022. “Tumor Metabolite Lactate Promotes Tumorigenesis by Modulating MOESIN Lactylation and Enhancing TGF‐Beta Signaling in Regulatory T Cells.” Cell Reports 39: 110986. 10.1016/j.celrep.2022.110986. 35732125

[acel70510-bib-0047] Gudgeon, N. , H. Munford , E. L. Bishop , et al. 2022. “Succinate Uptake by T Cells Suppresses Their Effector Function via Inhibition of Mitochondrial Glucose Oxidation.” Cell Reports 40: 111193. 10.1016/j.celrep.2022.111193. 35977513 PMC9638018

[acel70510-bib-0048] Guo, Z. , G. Wang , B. Wu , et al. 2020. “DCAF1 Regulates Treg Senescence via the ROS Axis During Immunological Aging.” Journal of Clinical Investigation 130: 5893–5908. 10.1172/JCI136466. 32730228 PMC7598062

[acel70510-bib-0049] Hakim, F. T. , F. A. Flomerfelt , M. Boyiadzis , and R. E. Gress . 2004. “Aging, Immunity and Cancer.” Current Opinion in Immunology 16: 151–156. 10.1016/j.coi.2004.01.009. 15023406

[acel70510-bib-0050] Han, A. , T. Peng , Y. Xie , et al. 2023. “Mitochondrial‐Regulated Tregs: Potential Therapeutic Targets for Autoimmune Diseases of the Central Nervous System.” Frontiers in Immunology 14: 1301074. 10.3389/fimmu.2023.1301074. 38149252 PMC10749924

[acel70510-bib-0051] Han, S. , P. Georgiev , A. E. Ringel , A. H. Sharpe , and M. C. Haigis . 2023. “Age‐Associated Remodeling of T Cell Immunity and Metabolism.” Cell Metabolism 35: 36–55. 10.1016/j.cmet.2022.11.005. 36473467 PMC10799654

[acel70510-bib-0052] Harber, K. J. , K. E. de Goede , S. G. S. Verberk , et al. 2020. “Succinate Is an Inflammation‐ Induced Immunoregulatory Metabolite in Macrophages.” Metabolites 10: 372. 10.3390/metabo10090372. 32942769 PMC7569821

[acel70510-bib-0053] Harman, D. 1991. “The Aging Process: Major Risk Factor for Disease and Death.” Proceedings of the National Academy of Sciences of the United States of America 88: 5360–5363. 10.1073/pnas.88.12.5360. 2052612 PMC51872

[acel70510-bib-0054] Harper, E. I. , T. S. Hilliard , E. F. Sheedy , et al. 2022. “Another Wrinkle With Age: Aged Collagen and Intra‐Peritoneal Metastasis of Ovarian Cancer.” Aging Cancer 3: 116–129. 10.1002/aac2.12049. 36188490 PMC9518742

[acel70510-bib-0055] Harper, E. I. , E. F. Sheedy , and M. S. Stack . 2018. “With Great Age Comes Great Metastatic Ability: Ovarian Cancer and the Appeal of the Aging Peritoneal Microenvironment.” Cancers 10: 230. 10.3390/cancers10070230. 29996539 PMC6070816

[acel70510-bib-0056] He, N. , W. Fan , B. Henriquez , et al. 2017. “Metabolic Control of Regulatory T Cell (Treg) Survival and Function by Lkb1.” Proceedings of the National Academy of Sciences of the United States of America 114: 12542–12547. 10.1073/pnas.1715363114. 29109251 PMC5703326

[acel70510-bib-0057] Hibino, S. , T. Kawazoe , H. Kasahara , et al. 2021. “Inflammation‐Induced Tumorigenesis and Metastasis.” International Journal of Molecular Sciences 22: 5421. 10.3390/ijms22115421. 34063828 PMC8196678

[acel70510-bib-0058] Hightower, R. D. , H. N. Nguyen , H. E. Averette , W. Hoskins , T. Harrison , and A. Steren . 1994. “National Survey of Ovarian Carcinoma. IV: Patterns of Care and Related Survival for Older Patients.” Cancer 73: 377–383. 8293403 10.1002/1097-0142(19940115)73:2<377::aid-cncr2820730223>3.0.co;2-#

[acel70510-bib-0059] Hoeijmakers, J. H. 2009. “DNA Damage, Aging, and Cancer.” New England Journal of Medicine 361: 1475–1485. 10.1056/NEJMra0804615. 19812404

[acel70510-bib-0060] Hou, X. , Y. Zhai , K. Hu , et al. 2022. “Aging Accelerates While Multiparity Delays Tumorigenesis in Mouse Models of High‐Grade Serous Carcinoma.” Gynecologic Oncology 165: 552–559. 10.1016/j.ygyno.2022.03.030. 35414426 PMC9533776

[acel70510-bib-0061] Huang, R. Y. , C. Eppolito , S. Lele , P. Shrikant , J. Matsuzaki , and K. Odunsi . 2015. “LAG3 and PD1 Co‐Inhibitory Molecules Collaborate to Limit CD8+ T Cell Signaling and Dampen Antitumor Immunity in a Murine Ovarian Cancer Model.” Oncotarget 6: 27359–27377. 10.18632/oncotarget.4751. 26318293 PMC4694995

[acel70510-bib-0063] Hurez, V. , B. J. Daniel , L. Sun , et al. 2012. “Mitigating Age‐Related Immune Dysfunction Heightens the Efficacy of Tumor Immunotherapy in Aged Mice.” Cancer Research 72: 2089–2099. 10.1158/0008-5472.CAN-11-3019. 22496463 PMC3328641

[acel70510-bib-0064] Isola, J. V. V. , J. D. Hense , C. A. P. Osorio , et al. 2024. “Reproductive Ageing: Inflammation, Immune Cells, and Cellular Senescence in the Aging Ovary.” Reproduction (Cambridge, England) 168: e230499. 10.1530/REP-23-0499. 38744316 PMC11301429

[acel70510-bib-0065] Jagger, A. , Y. Shimojima , J. J. Goronzy , and C. M. Weyand . 2014. “Regulatory T Cells and the Immune Aging Process: A Mini‐Review.” Gerontology 60: 130–137. 10.1159/000355303. 24296590 PMC4878402

[acel70510-bib-0066] Jarnicki, A. G. , J. Lysaght , S. Todryk , and K. H. Mills . 2006. “Suppression of Antitumor Immunity by IL‐10 and TGF‐Beta‐Producing T Cells Infiltrating the Growing Tumor: Influence of Tumor Environment on the Induction of CD4+ and CD8+ Regulatory T Cells.” Journal of Immunology 177: 896–904. 10.4049/jimmunol.177.2.896. 16818744

[acel70510-bib-0067] Kalim, K. W. , J. Q. Yang , M. Wunderlich , et al. 2022. “Targeting of Cdc42 GTPase in Regulatory T Cells Unleashes Antitumor T‐Cell Immunity.” Journal for Immunotherapy of Cancer 10: e004806. 10.1136/jitc-2022-004806. 36427906 PMC9703354

[acel70510-bib-0068] Kamada, T. , Y. Togashi , C. Tay , et al. 2019. “PD‐1(+) Regulatory T Cells Amplified by PD‐1 Blockade Promote Hyperprogression of Cancer.” Proceedings of the National Academy ofSciences of the United States of America 116: 9999–10008. 10.1073/pnas.1822001116. PMC6525547 31028147

[acel70510-bib-0069] Kempkes, R. W. M. , I. Joosten , H. Koenen , and X. He . 2019. “Metabolic Pathways Involved in Regulatory T Cell Functionality.” Frontiers in Immunology 10: 2839. 10.3389/fimmu.2019.02839. 31849995 PMC6902900

[acel70510-bib-0070] Khan, H. Y. , M. Kamgar , A. Aboukameel , et al. 2023. “Targeting Cellular Metabolism With CPI‐ 613 Sensitizes Pancreatic Cancer Cells to Radiation Therapy.” Advances in Radiation Oncology 8: 101122. 10.1016/j.adro.2022.101122. 36479231 PMC9720358

[acel70510-bib-0071] Kim, J. H. , B. S. Kim , and S. K. Lee . 2020. “Regulatory T Cells in Tumor Microenvironment and Approach for Anticancer Immunotherapy.” Immune Network 20: e4. 10.4110/in.2020.20.e4. 32158592 PMC7049587

[acel70510-bib-0072] Kinsella, S. , C. W. Smith , K. McKenna , et al. 2023. “Elevated Metabolite Secretion Facilitates a Treg‐Mediated Immunosuppressive Microenvironment in the Solid Tumor.” Blood 142: 3458. 10.1182/blood-2023-189868.

[acel70510-bib-0073] Kolben, T. , M. Mannewitz , C. Perleberg , et al. 2022. “Presence of Regulatory T‐Cells in Endometrial Cancer Predicts Poorer Overall Survival and Promotes Progression of Tumor Cells.” Cellular Oncology (Dordrecht) 45: 1171–1185. 10.1007/s13402-022-00708-2. PMC9747805 36098901

[acel70510-bib-0074] Kouidhi, S. , A. B. Elgaaied , and S. Chouaib . 2017. “Impact of Metabolism on T‐Cell Differentiation and Function and Cross Talk With Tumor Microenvironment.” Frontiers in Immunology 8: 270. 10.3389/fimmu.2017.00270. 28348562 PMC5346542

[acel70510-bib-0075] Kuilman, T. , C. Michaloglou , L. C. Vredeveld , et al. 2008. “Oncogene‐Induced Senescence Relayed by an Interleukin‐Dependent Inflammatory Network.” Cell 133: 1019–1031. 10.1016/j.cell.2008.03.039. 18555778

[acel70510-bib-0076] Langmar, Z. , and S. Csomor . 2006. “Treatment of Epithelial Ovarian Cancer [in Hungarian].” Orvosi Hetilap 147: 1627–1632. 17017677

[acel70510-bib-0077] Li, C. , P. Jiang , S. Wei , X. Xu , and J. Wang . 2020. “Regulatory T Cells in Tumor Microenvironment: New Mechanisms, Potential Therapeutic Strategies and Future Prospects.” Molecular Cancer 19: 116. 10.1186/s12943-020-01234-1. 32680511 PMC7367382

[acel70510-bib-0078] Li, M. , D. Yao , X. Zeng , et al. 2019. “Age Related Human T Cell Subset Evolution and Senescence.” Immunity & Ageing 16: 24. 10.1186/s12979-019-0165-8. 31528179 PMC6739976

[acel70510-bib-0079] Li, R. , J. Xu , M. Wu , et al. 2023. “Circulating CD4(+) Treg, CD8(+) Treg, and CD3(+) Gammadelta T Cell Subpopulations in Ovarian Cancer.” Medicina (Kaunas, Lithuania) 59: 205. 10.3390/medicina59020205. 36837407 PMC9958753

[acel70510-bib-0080] Li, X. , C. Li , W. Zhang , Y. Wang , P. Qian , and H. Huang . 2023. “Inflammation and Aging: Signaling Pathways and Intervention Therapies.” Signal Transduction and Targeted Therapy 8: 239. 10.1038/s41392-023-01502-8. 37291105 PMC10248351

[acel70510-bib-0081] Li, Y. , P. Guaman Tipan , H. J. Selden , J. Srinivasan , L. P. Hale , and L. I. R. Ehrlich . 2023. “CCR4 and CCR7 Differentially Regulate Thymocyte Localization With Distinct Outcomes for Central Tolerance.” eLife 12: e80443. 10.7554/eLife.80443. 37266571 PMC10325719

[acel70510-bib-0082] Liao, J. B. , K. J. Ovenell , E. E. Curtis , et al. 2015. “Preservation of Tumor‐Host Immune Interactions With Luciferase‐Tagged Imaging in a Murine Model of Ovarian Cancer.” Journal for Immunotherapy of Cancer 3: 16. 10.1186/s40425-015-0060-6. 25992288 PMC4437454

[acel70510-bib-0083] Liu, N. , J. Zhang , M. Yan , et al. 2023. “Supplementation With Alpha‐Ketoglutarate Improved the Efficacy of Anti‐PD1 Melanoma Treatment Through Epigenetic Modulation of PD‐L1.” Cell Death & Disease 14: 170. 10.1038/s41419-023-05692-5. 36854755 PMC9974984

[acel70510-bib-0084] Liu, S. , S. Liao , L. Liang , J. Deng , and Y. Zhou . 2023. “The Relationship Between CD4(+) T Cell Glycolysis and Their Functions.” Trends in Endocrinology and Metabolism 34: 345–360. 10.1016/j.tem.2023.03.006. 37061430

[acel70510-bib-0085] Liu, X. , C. L. Hartman , L. Li , et al. 2021. “Reprogramming Lipid Metabolism Prevents Effector T Cell Senescence and Enhances Tumor Immunotherapy.” Science Translational Medicine 13: eaaz6314. 10.1126/scitranslmed.aaz6314. 33790024 PMC12040281

[acel70510-bib-0086] Liu, X. , W. Mo , J. Ye , et al. 2018. “Regulatory T Cells Trigger Effector T Cell DNA Damage and Senescence Caused by Metabolic Competition.” Nature Communications 9: 249. 10.1038/s41467-017-02689-5. PMC5770447 29339767

[acel70510-bib-0087] Loughran, E. A. , A. K. Leonard , T. S. Hilliard , et al. 2018. “Aging Increases Susceptibility to Ovarian Cancer Metastasis in Murine Allograft Models and Alters Immune Composition of Peritoneal Adipose Tissue.” Neoplasia 20: 621–631. 10.1016/j.neo.2018.03.007. 29754071 PMC5994778

[acel70510-bib-0088] Ma, S. , Y. Ming , J. Wu , and G. Cui . 2024. “Cellular Metabolism Regulates the Differentiation and Function of T‐Cell Subsets.” Cellular & Molecular Immunology 21: 419–435. 10.1038/s41423-024-01148-8. 38565887 PMC11061161

[acel70510-bib-0089] Mandilaras, V. , S. Garg , M. Cabanero , et al. 2019. “TP53 Mutations in High Grade Serous Ovarian Cancer and Impact on Clinical Outcomes: A Comparison of Next Generation Sequencing and Bioinformatics Analyses.” International Journal of Gynecological Cancer 29: 346–352. 10.1136/ijgc-2018-000087. 30659026

[acel70510-bib-0090] Mangali, S. , A. Bhat , M. P. Udumula , I. Dhar , D. Sriram , and A. Dhar . 2019. “Inhibition of Protein Kinase R Protects Against Palmitic Acid‐Induced Inflammation, Oxidative Stress, and Apoptosis Through the JNK/NF‐kB/NLRP3 Pathway in Cultured H9C2 Cardiomyocytes.” Journal of Cellular Biochemistry 120: 3651–3663. 10.1002/jcb.27643. 30259999

[acel70510-bib-0092] Moon, Y. W. , J. Hajjar , P. Hwu , and A. Naing . 2015. “Targeting the Indoleamine 2,3‐Dioxygenase Pathway in Cancer.” Journal for Immunotherapy of Cancer 3, no. 51: 9. 10.1186/s40425-015-0094-9. 26674411 PMC4678703

[acel70510-bib-0093] Moreno Ayala, M. A. , Z. Li , and M. DuPage . 2019. “Treg Programming and Therapeutic Reprogramming in Cancer.” Immunology 157: 198–209. 10.1111/imm.13058. 30866047 PMC6587317

[acel70510-bib-0094] Mortazavi Farsani, S. S. , and V. Verma . 2023. “Lactate Mediated Metabolic Crosstalk Between Cancer and Immune Cells and Its Therapeutic Implications.” Frontiers in Oncology 13: 1175532. 10.3389/fonc.2023.1175532. 37234972 PMC10206240

[acel70510-bib-0095] Moskalev, A. , I. Stambler , and C. Caruso . 2020. “Innate and Adaptive Immunity in Aging and Longevity: The Foundation of Resilience.” Aging and Disease 11: 1363–1373. 10.14336/AD.2020.0603. 33269094 PMC7673842

[acel70510-bib-0096] Narita, M. , and S. W. Lowe . 2005. “Senescence Comes of Age.” Nature Medicine 11: 920–922. 10.1038/nm0905-920. 16145569

[acel70510-bib-0097] National Cancer Institute, & Surveillance Epidemiology and End Results Program . 2023. “Ovary: SEER 5‐Year Relative Survival Rates, 2015–2021.” Female by Age, All Races/Ethnicities, All Stages. https://seer.cancer.gov/statistics‐network/explorer/application.html?site=61&data_type=4&graph_type=5&compareBy=age_range&chk_age_range_9=9&chk_age_range_141=141&chk_age_range_157=157&series=9&hdn_sex=3&race=1&stage=101&advopt_precision=1&advopt_show_ci=on&hdn_view=0&advopt_show_apc=on&advopt_display=2#resultsRegion0.

[acel70510-bib-0098] Ohue, Y. , and H. Nishikawa . 2019. “Regulatory T (Treg) Cells in Cancer: Can Treg Cells Be a New Therapeutic Target?” Cancer Science 110: 2080–2089. 10.1111/cas.14069. 31102428 PMC6609813

[acel70510-bib-0099] Ou, H. L. , and B. Schumacher . 2018. “DNA Damage Responses and p53 in the Aging Process.” Blood 131: 488–495. 10.1182/blood-2017-07-746396. 29141944 PMC6839964

[acel70510-bib-0100] Palatella, M. , S. M. Guillaume , M. A. Linterman , and J. Huehn . 2022. “The Dark Side of Tregs During Aging.” Frontiers in Immunology 13: 940705. 10.3389/fimmu.2022.940705. 36016952 PMC9398463

[acel70510-bib-0101] Phan, A. T. , and A. W. Goldrath . 2015. “Hypoxia‐Inducible Factors Regulate T Cell Metabolism and Function.” Molecular Immunology 68: 527–535. 10.1016/j.molimm.2015.08.004. 26298577 PMC4679538

[acel70510-bib-0102] Ramello, M. C. , N. G. Nunez , J. Tosello Boari , et al. 2021. “Polyfunctional KLRG‐ 1(+)CD57(+) Senescent CD4(+) T Cells Infiltrate Tumors and Are Expanded in Peripheral Blood From Breast Cancer Patients.” Frontiers in Immunology 12: 713132. 10.3389/fimmu.2021.713132. 34386013 PMC8353459

[acel70510-bib-0103] Reichard, A. , and K. Asosingh . 2019. “Best Practices for Preparing a Single Cell Suspension From Solid Tissues for Flow Cytometry.” Cytometry. Part A 95: 219–226. 10.1002/cyto.a.23690. PMC6375754 30523671

[acel70510-bib-0104] Reina‐Campos, M. , N. E. Scharping , and A. W. Goldrath . 2021. “CD8(+) T Cell Metabolism in Infection and Cancer.” Nature Reviews. Immunology 21: 718–738. 10.1038/s41577-021-00537-8. PMC8806153 33981085

[acel70510-bib-0143] Rhoads, T. W. , and R. M. Anderson . 2020. “Alpha‐Ketoglutarate, the Metabolite That Regulates Aging in Mice.” Cell Metabolism 32, no. 3: 323–325. 32877686 10.1016/j.cmet.2020.08.009 PMC8191137

[acel70510-bib-0105] Roby, K. F. , C. C. Taylor , J. P. Sweetwood , et al. 2000. “Development of a Syngeneic Mouse Model for Events Related to Ovarian Cancer.” Carcinogenesis 21: 585–591. 10.1093/carcin/21.4.585. 10753190

[acel70510-bib-0106] Rodriguez, I. J. , N. Lalinde Ruiz , M. Llano Leon , et al. 2020. “Immunosenescence Study of T Cells: A Systematic Review.” Frontiers in Immunology 11: 604591. 10.3389/fimmu.2020.604591. 33519813 PMC7843425

[acel70510-bib-0107] Salam, N. , S. Rane , R. Das , et al. 2013. “T Cell Ageing: Effects of Age on Development, Survival & Function.” Indian Journal of Medical Research 138: 595–608. 24434315 PMC3928693

[acel70510-bib-0142] Sandalova, E. , J. Goh , Z. X. Lim , et al. 2023. “Alpha‐Ketoglutarate Supplementation and BiologicaL agE in Middle‐Aged Adults (ABLE)—Intervention Study Protocol.” Geroscience 45, no. 5: 2897–2907. 37217632 10.1007/s11357-023-00813-6 PMC10643463

[acel70510-bib-0108] Schmidt, A. , N. Oberle , and P. H. Krammer . 2012. “Molecular Mechanisms of Treg‐Mediated T Cell Suppression.” Frontiers in Immunology 3: 51. 10.3389/fimmu.2012.00051. 22566933 PMC3341960

[acel70510-bib-0109] Sedrak, M. S. , and H. J. Cohen . 2023. “The Aging‐Cancer Cycle: Mechanisms and Opportunities for Intervention.” Journals of Gerontology. Series A, Biological Sciences and Medical Sciences 78: 1234–1238. 10.1093/gerona/glac247. 36512079 PMC10329223

[acel70510-bib-0144] Senkus, K. E. , K. M. Crowe‐White , A. C. Bolland , J. L. Locher , and J. D. Ard . 2022. “Changes in Adiponectin: Leptin Ratio Among Older Adults with Obesity Following a 12‐Month Exercise and Diet Intervention.” Nutrition & Diabetes 12, no. 1: 30. 35654771 10.1038/s41387-022-00207-1 PMC9163185

[acel70510-bib-0110] Shi, L. Z. , R. Wang , G. Huang , et al. 2011. “HIF1alpha‐Dependent Glycolytic Pathway Orchestrates a Metabolic Checkpoint for the Differentiation of TH17 and Treg Cells.” Journal of Experimental Medicine 208: 1367–1376. 10.1084/jem.20110278. 21708926 PMC3135370

[acel70510-bib-0111] Shim, S. H. , M. C. Lim , D. Lee , et al. 2022. “Cause‐Specific Mortality Rate of Ovarian Cancer in the Presence of Competing Risks of Death: A Nationwide Population‐Based Cohort Study.” Journal of Gynecologic Oncology 33: e5. 10.3802/jgo.2022.33.e5. 34783208 PMC8728665

[acel70510-bib-0112] Sia, T. Y. , W. P. Tew , C. Purdy , et al. 2023. “The Effect of Older Age on Treatment Outcomes in Women With Advanced Ovarian Cancer Receiving Chemotherapy: An NRG‐Oncology/Gynecologic Oncology Group (GOG‐0182‐ICON5) Ancillary Study.” Gynecologic Oncology 173: 130–137. 10.1016/j.ygyno.2023.03.018. 37148580 PMC10414765

[acel70510-bib-0113] Simon, S. , and N. Labarriere . 2017. “PD‐1 Expression on Tumor‐Specific T Cells: Friend or Foe for Immunotherapy?” Oncoimmunology 7: e1364828. 10.1080/2162402X.2017.1364828. 29296515 PMC5739549

[acel70510-bib-0114] Sitnikova, S. I. , J. A. Walker , L. B. Prickett , et al. 2023. “Age‐Induced Changes in Anti‐Tumor Immunity Alter the Tumor Immune Infiltrate and Impact Response to Immuno‐Oncology Treatments.” Frontiers in Immunology 14: 1258291. 10.3389/fimmu.2023.1258291. 37920465 PMC10618668

[acel70510-bib-0115] So, L. , K. Obata‐Ninomiya , A. Hu , et al. 2023. “Regulatory T Cells Suppress CD4+ Effector T Cell Activation by Controlling Protein Synthesis.” Journal of Experimental Medicine 220: e20221676. 10.1084/jem.20221676. 36598533 PMC9827529

[acel70510-bib-0116] Tew, W. P. 2016. “Ovarian Cancer in the Older Woman.” Journal of Geriatric Oncology 7: 354–361. 10.1016/j.jgo.2016.07.008. 27499341

[acel70510-bib-0117] Thigpen, T. , M. F. Brady , G. A. Omura , et al. 1993. “Age as a Prognostic Factor in Ovarian Carcinoma. The Gynecologic Oncology Group Experience.” Cancer 71: 606–614. 10.1002/cncr.2820710218. 8420683

[acel70510-bib-0118] Torre, L. A. , B. Trabert , C. E. DeSantis , et al. 2018. “Ovarian Cancer Statistics, 2018.” CA: a Cancer Journal for Clinicians 68: 284–296. 10.3322/caac.21456. 29809280 PMC6621554

[acel70510-bib-0119] Tortorella, L. , G. Vizzielli , D. Fusco , et al. 2017. “Ovarian Cancer Management in the Oldest Old: Improving Outcomes and Tailoring Treatments.” Aging and Disease 8: 677–684. 10.14336/AD.2017.0607. 28966809 PMC5614329

[acel70510-bib-0120] Tran, K. A. , C. M. Dillingham , and R. Sridharan . 2019. “The Role of Alpha‐Ketoglutarate‐ Dependent Proteins in Pluripotency Acquisition and Maintenance.” Journal of Biological Chemistry 294: 5408–5419. 10.1074/jbc.TM118.000831. 30181211 PMC6462505

[acel70510-bib-0121] Trauelsen, M. , T. K. Hiron , D. Lin , et al. 2021. “Extracellular Succinate Hyperpolarizes M2 Macrophages Through SUCNR1/GPR91‐Mediated Gq Signaling.” Cell Reports 35: 109246. 10.1016/j.celrep.2021.109246. 34133934

[acel70510-bib-0122] Udumula, M. P. , L. M. Poisson , I. Dutta , et al. 2022. “Divergent Metabolic Effects of Metformin Merge to Enhance Eicosapentaenoic Acid Metabolism and Inhibit Ovarian Cancer In Vivo.” Cancers 14: 1504. 10.3390/cancers14061504. 35326656 PMC8946838

[acel70510-bib-0123] Udumula, M. P. , F. Rashid , H. Singh , et al. 2024. “Targeting Mitochondrial Metabolism With CPI‐613 in Chemoresistant Ovarian Tumors.” Journal of Ovarian Research 17: 226. 10.1186/s13048-024-01546-6. 39543742 PMC11566742

[acel70510-bib-0124] Udumula, M. P. , S. Sakr , S. Dar , et al. 2021. “Ovarian Cancer Modulates the Immunosuppressive Function of CD11b(+)Gr1(+) Myeloid Cells via Glutamine Metabolism.” Molecular Metabolism 53: 101272. 10.1016/j.molmet.2021.101272. 34144215 PMC8267600

[acel70510-bib-0125] Udumula, M. P. , H. Singh , F. Rashid , et al. 2023. “Intermittent Fasting Induced Ketogenesis Inhibits Mouse Epithelial Ovarian Cancer by Promoting Antitumor T Cell Response.” iScience 26: 107839. 10.1016/j.isci.2023.107839. 37822507 PMC10562806

[acel70510-bib-0126] Walton, J. , J. Blagih , D. Ennis , et al. 2016. “CRISPR/Cas9‐Mediated Trp53 and Brca2 Knockout to Generate Improved Murine Models of Ovarian High‐Grade Serous Carcinoma.” Cancer Research 76: 6118–6129. 10.1158/0008-5472.CAN-16-1272. 27530326 PMC5802386

[acel70510-bib-0127] Wang, H. , F. Franco , and P. C. Ho . 2017. “Metabolic Regulation of Tregs in Cancer: Opportunities for Immunotherapy.” Trends Cancer 3: 583–592. 10.1016/j.trecan.2017.06.005. 28780935

[acel70510-bib-0128] Won, E. , A. Hurria , T. Feng , et al. 2013. “CA125 Level Association With Chemotherapy Toxicity and Functional Status in Older Women With Ovarian Cancer.” International Journal of Gynecological Cancer 23: 1022–1028. 10.1097/IGC.0b013e318299438a. 23765208 PMC3772622

[acel70510-bib-0130] Yager, E. J. , M. Ahmed , K. Lanzer , T. D. Randall , D. L. Woodland , and M. A. Blackman . 2008. “Age‐Associated Decline in T Cell Repertoire Diversity Leads to Holes in the Repertoire and Impaired Immunity to Influenza Virus.” Journal of Experimental Medicine 205: 711–723. 10.1084/jem.20071140. 18332179 PMC2275391

[acel70510-bib-0131] Yan, Y. , L. Huang , Y. Liu , et al. 2022. “Metabolic Profiles of Regulatory T Cells and Their Adaptations to the Tumor Microenvironment: Implications for Antitumor Immunity.” Journal of Hematology & Oncology 15: 104. 10.1186/s13045-022-01322-3. 35948909 PMC9364625

[acel70510-bib-0132] Yang, K. Y. , J. Liao , Z. Ma , et al. 2024. “Single‐Cell Transcriptomics of Treg Reveals Hallmarks and Trajectories of Immunological Aging.” Journal of Leukocyte Biology 115: 19–35. 10.1093/jleuko/qiad104. 37675661

[acel70510-bib-0133] Ye, J. , X. Huang , E. C. Hsueh , et al. 2012. “Human Regulatory T Cells Induce T‐Lymphocyte Senescence.” Blood 120: 2021–2031. 10.1182/blood-2012-03-416040. 22723548 PMC3437594

[acel70510-bib-0134] Ye, L. L. , W. B. Peng , Y. R. Niu , et al. 2020. “Accumulation of TNFR2‐Expressing Regulatory T Cells in Malignant Pleural Effusion of Lung Cancer Patients Is Associated With Poor Prognosis.” Annals of Translational Medicine 8: 1647. 10.21037/atm-20-7181. 33490159 PMC7812164

[acel70510-bib-0135] Yigit, R. , L. F. Massuger , C. G. Figdor , and R. Torensma . 2010. “Ovarian Cancer Creates a Suppressive Microenvironment to Escape Immune Elimination.” Gynecologic Oncology 117: 366–372. 10.1016/j.ygyno.2010.01.019. 20144842

[acel70510-bib-0136] Zahoor, I. , M. Nematullah , M. E. Ahmed , et al. 2025. “Maresin‐1 Promotes Neuroprotection and Modulates Metabolic and Inflammatory Responses in Disease‐Associated Cell Types in Preclinical Models of Multiple Sclerosis.” Journal of Biological Chemistry 301: 108226. 10.1016/j.jbc.2025.108226. 39864620 PMC11903811

[acel70510-bib-0137] Zhang, S. , X. Ke , S. Zeng , et al. 2015. “Analysis of CD8+ Treg Cells in Patients With Ovarian Cancer: A Possible Mechanism for Immune Impairment.” Cellular & Molecular Immunology 12: 580–591. 10.1038/cmi.2015.57. 26166762 PMC4579658

[acel70510-bib-0138] Zhao, B. , B. Wu , N. Feng , et al. 2023. “Aging Microenvironment and Antitumor Immunity for Geriatric Oncology: The Landscape and Future Implications.” Journal of Hematology & Oncology Oncology 16, no. 28: 4. 10.1186/s13045-023-01426-4. PMC10032017 36945046

[acel70510-bib-0140] Zong, Y. , K. Deng , and W. P. Chong . 2024. “Regulation of Treg Cells by Cytokine Signaling and Co‐Stimulatory Molecules.” Frontiers in Immunology 15: 1387975. 10.3389/fimmu.2024.1387975. 38807592 PMC11131382

