# Supplementary material for: Aging‐Driven Immunosuppression: The Role of Tregs in the Ovarian Tumor Microenvironment
Source: Aging Cell. 2026 Apr 24;25(5):e70510. doi: 10.1111/acel.70510 (PMC13109645; doi:10.1111/acel.70510)

Supplementary figures

Figure S1: Aging exacerbates preclinical EOC models:

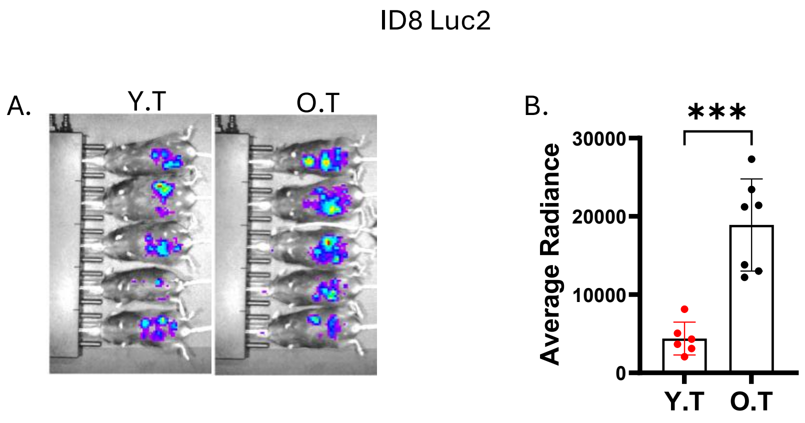

Figure S2: Aging enhances Senescence Associated Secretory Phenotype in EOC.

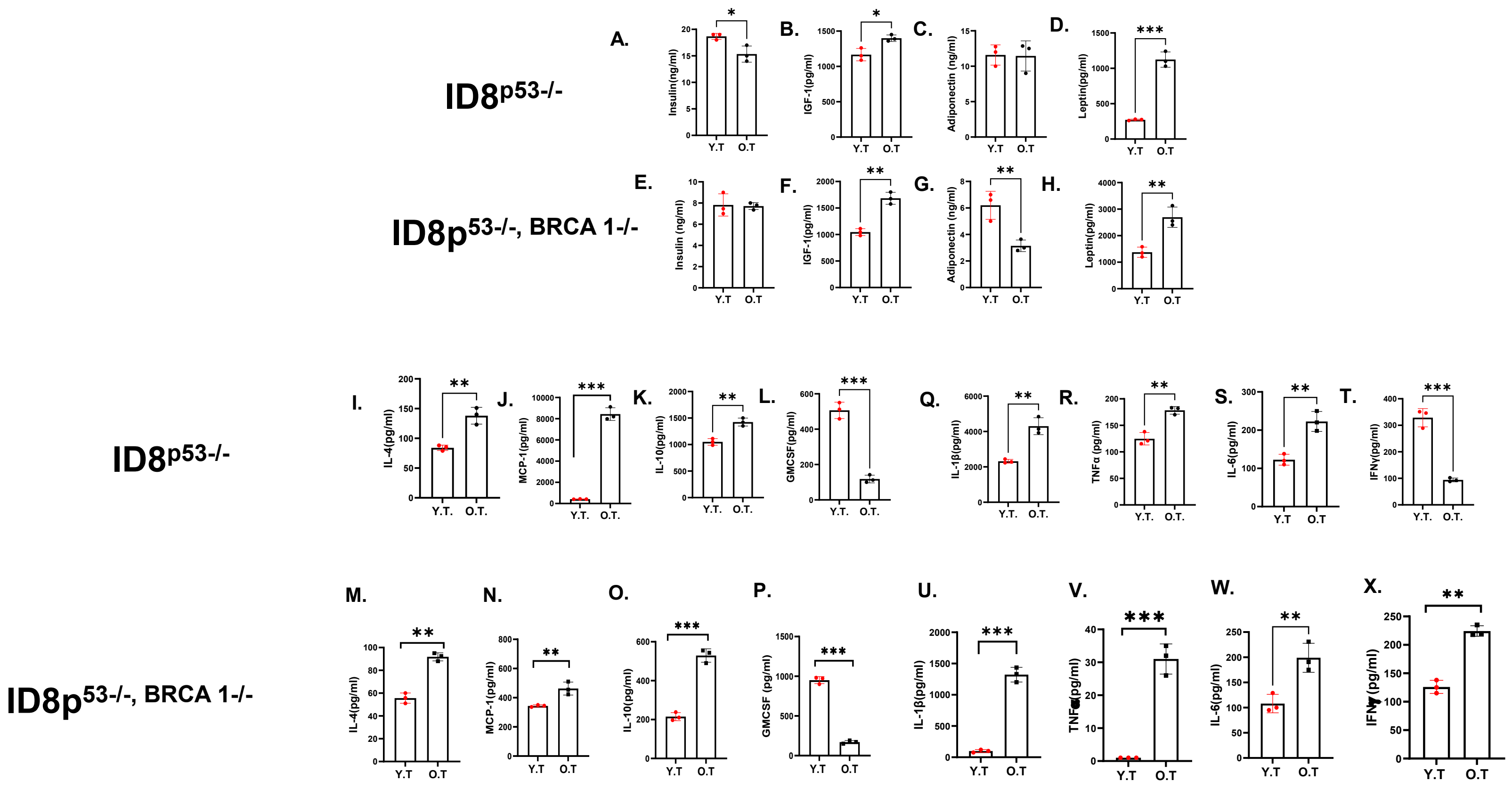

S3: Aging induces differential systemic immune responses in response to EOC

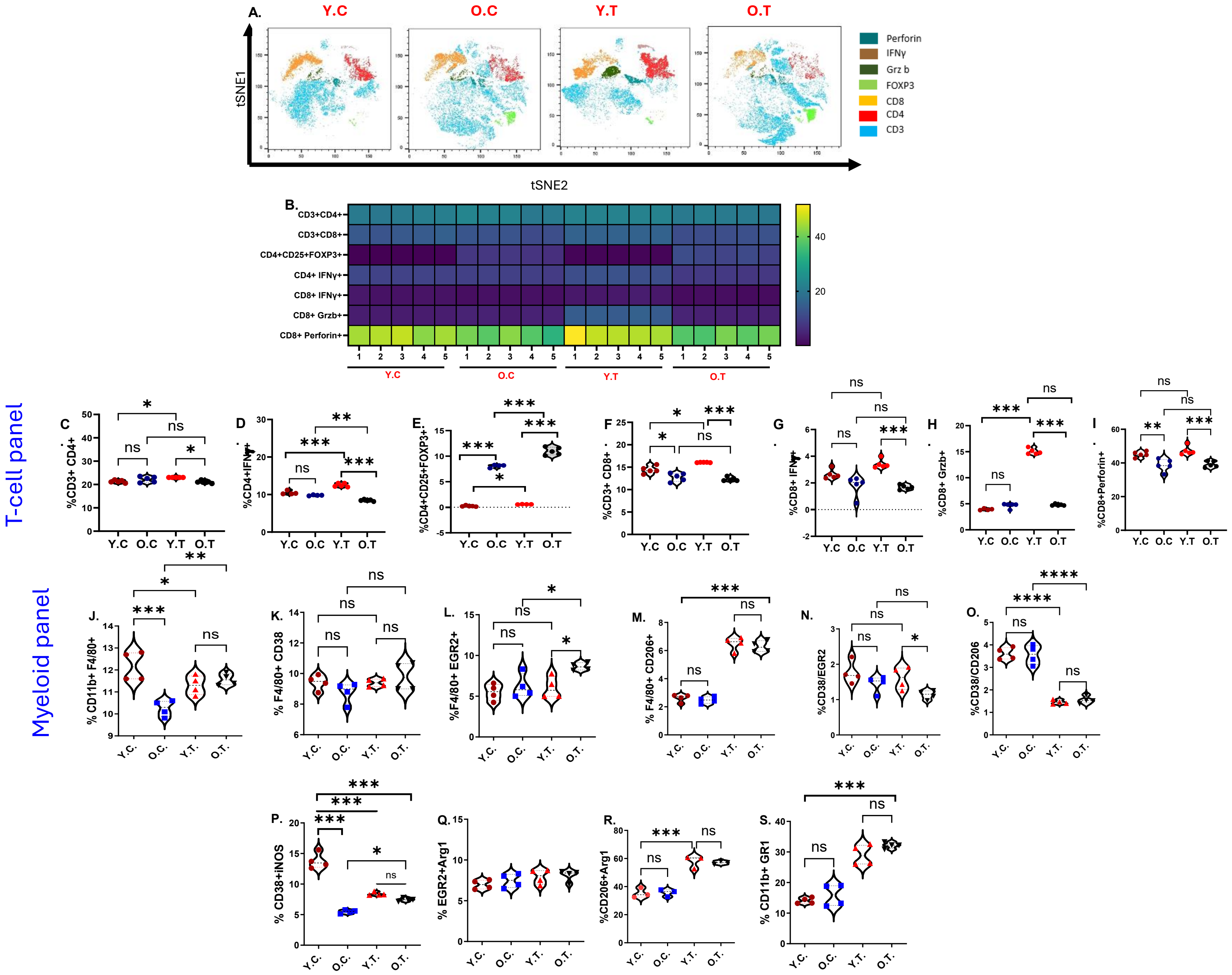

Figure S 4: Aging induces Differential T cell response in Ovarian TME.

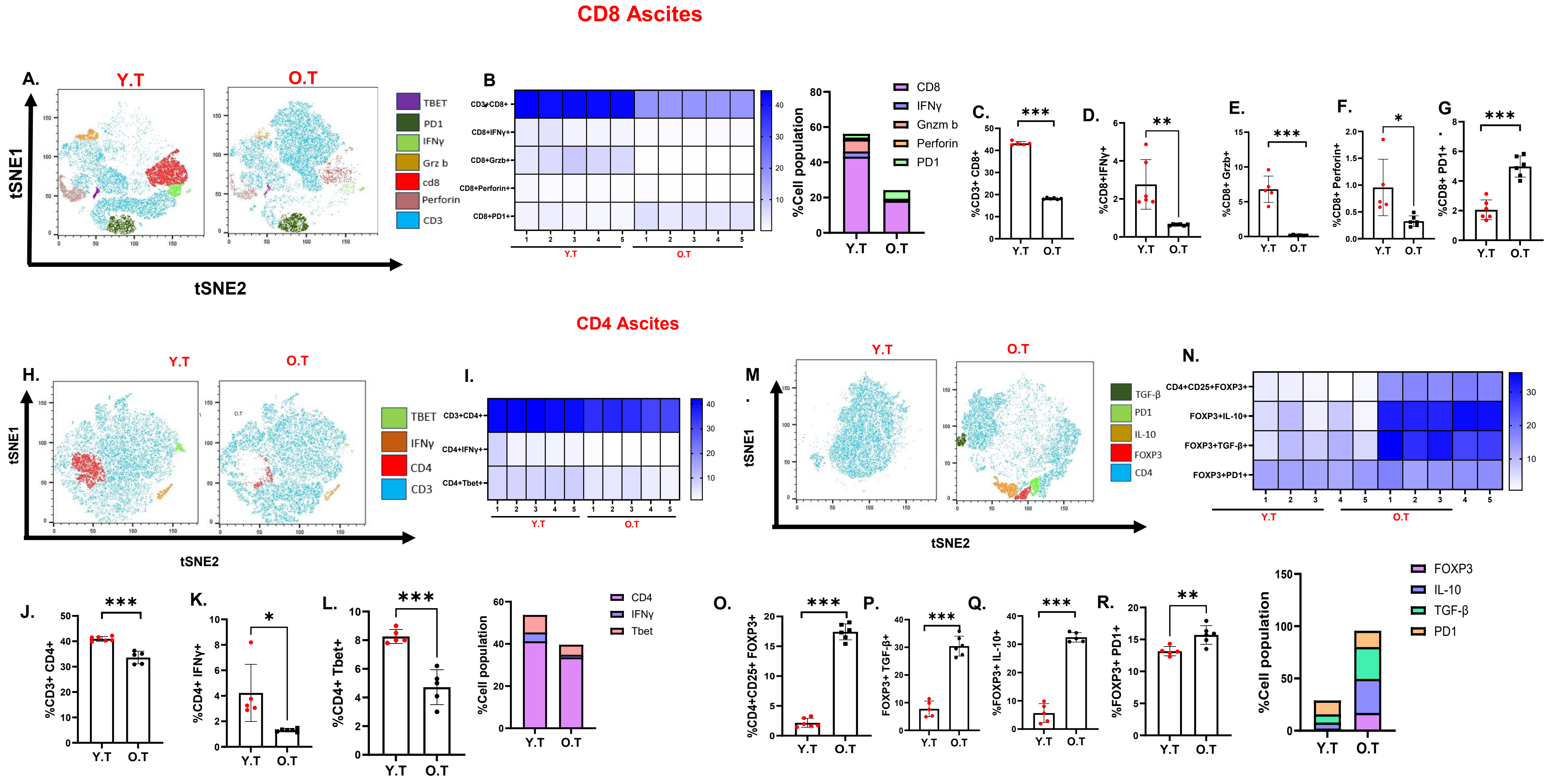

Figure S5: Aging diminishes the intratumor T cell response in ID8 BRCA1-/- injected EOC mice.

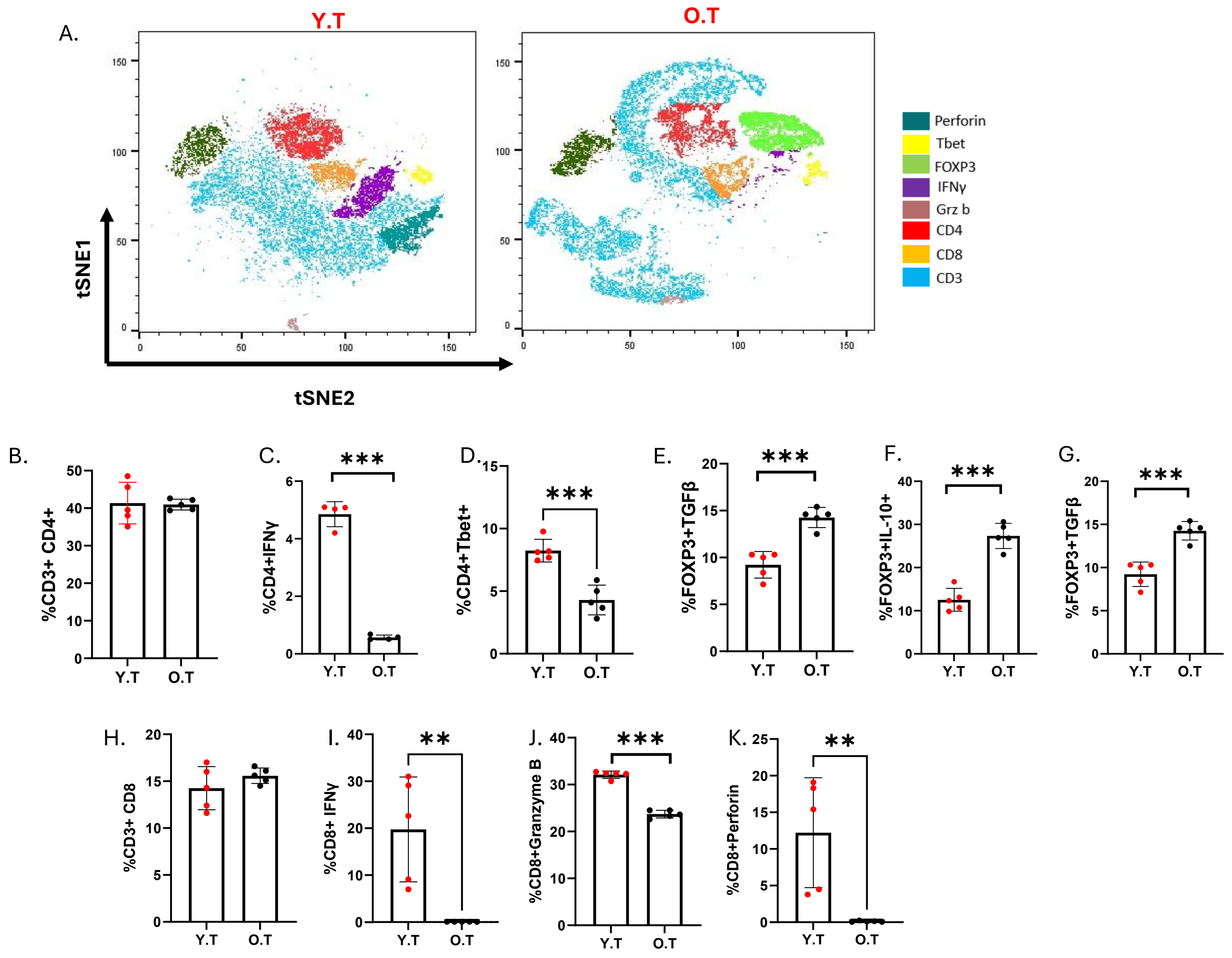

Figure S6: Aging diminishes the ascitic T cell response in ID8 BRCA1-/- injected EOC mice.

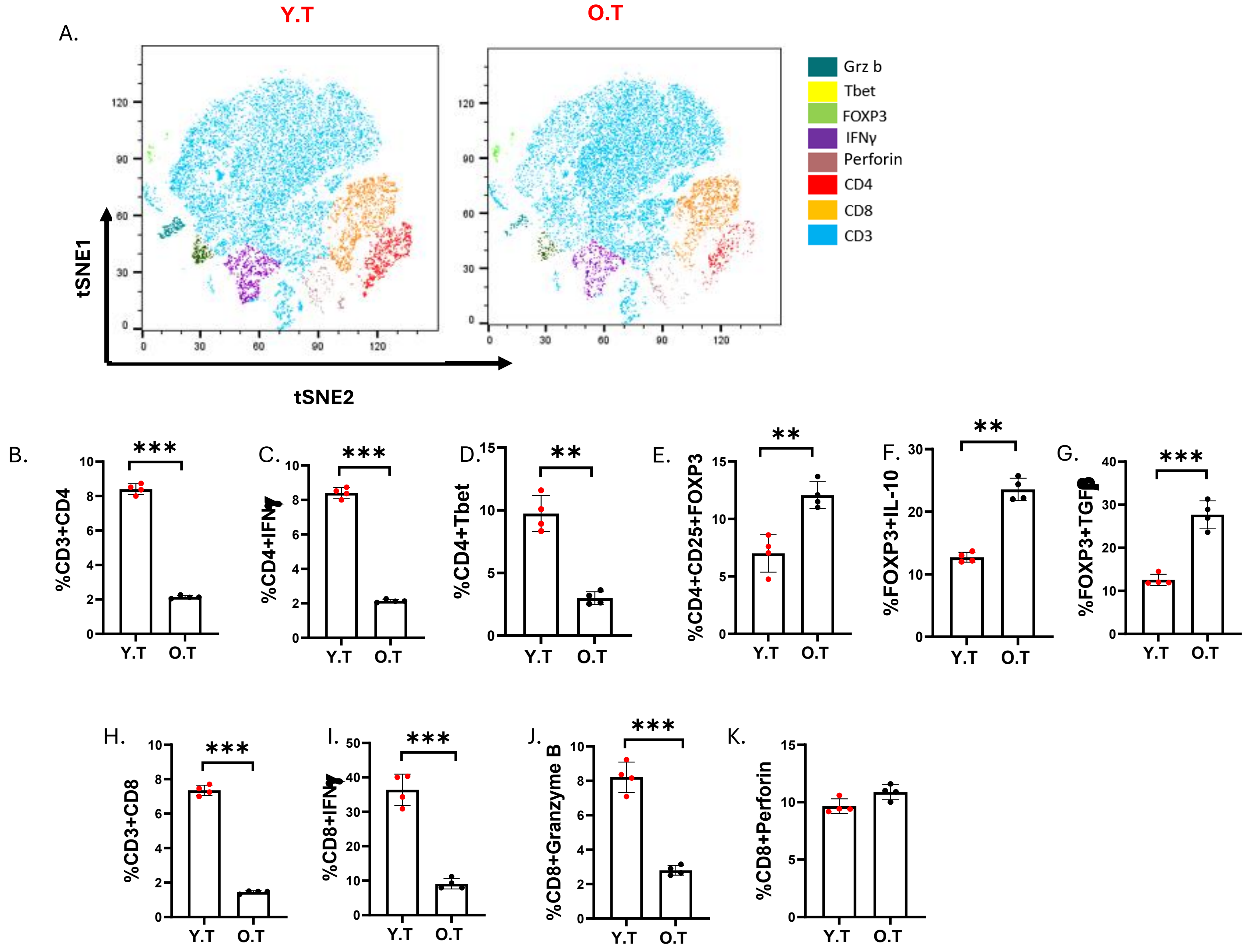

Figure S7: Treg depletion restores intratumoral antitumor immunity and reduces T-cell senescence in aged EOC

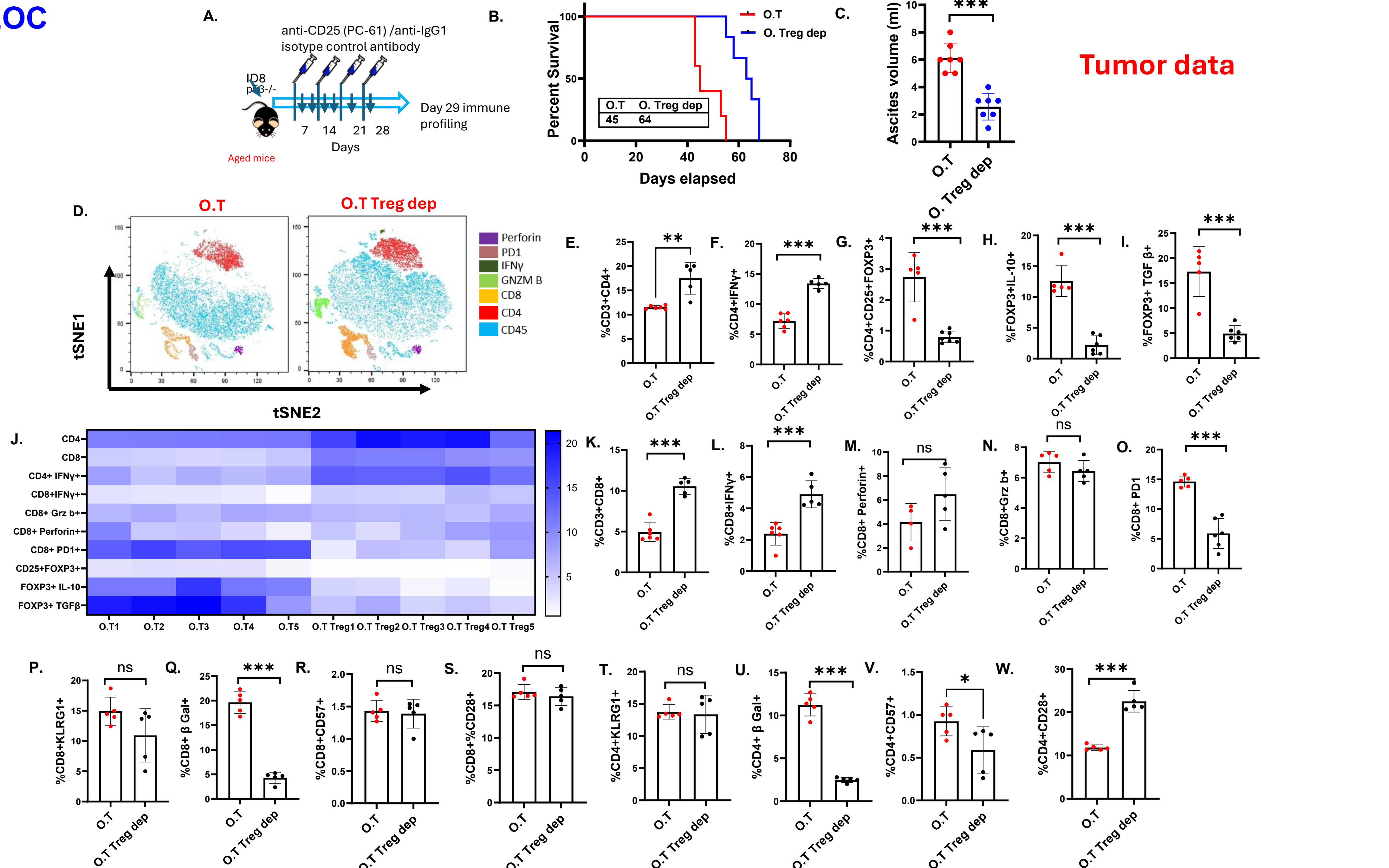

Figure S8: Treg depletion reprograms the ascitic immune microenvironment and enhances effector T-cell function in aged EOC.

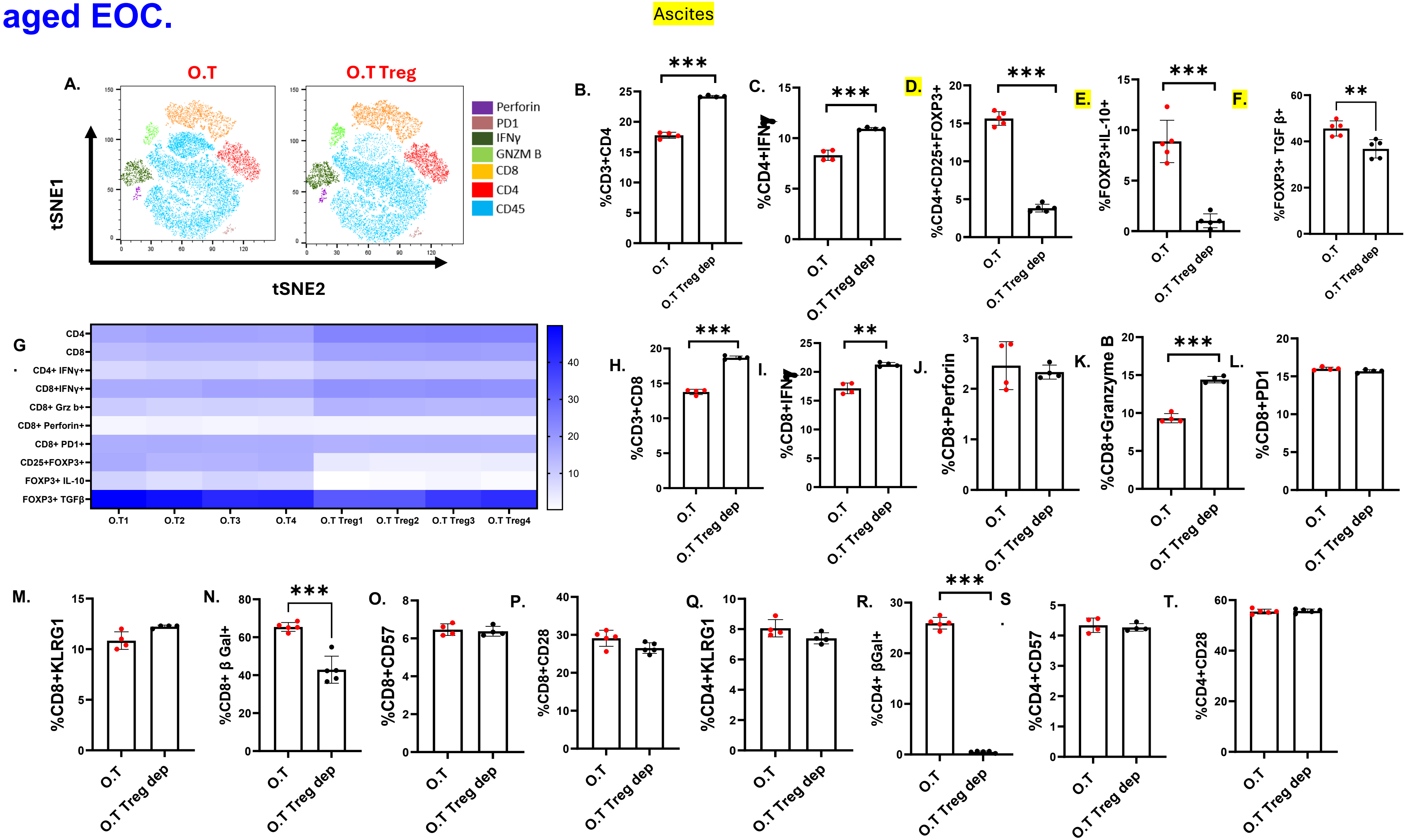

Fig S9: Treg depletion promotes systemic immune restoration and reverses T-cell dysfunction in aged EOC.

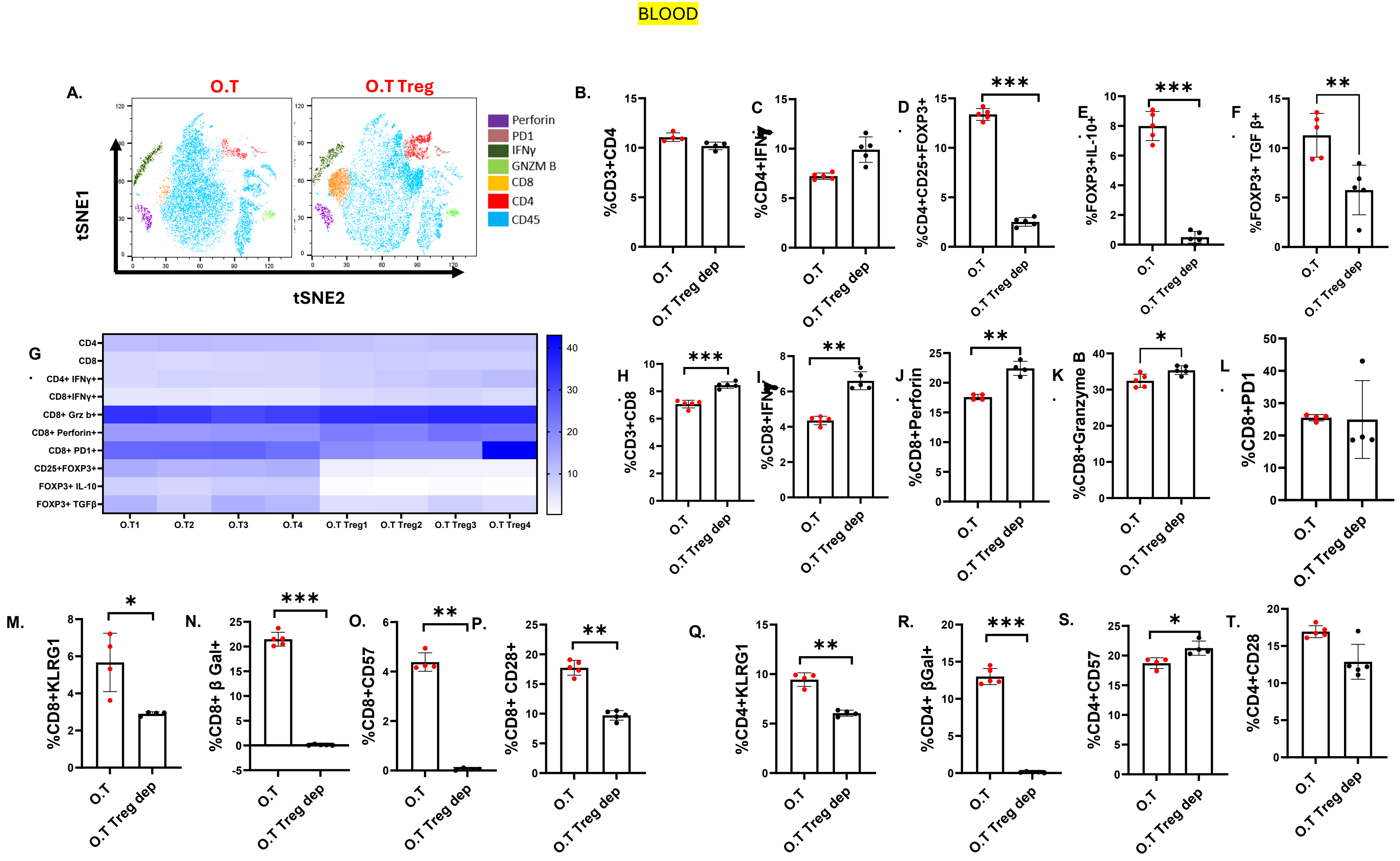

Fig S10: Tregs from aged EOC mice prefer OXPHOS for immunosuppression.

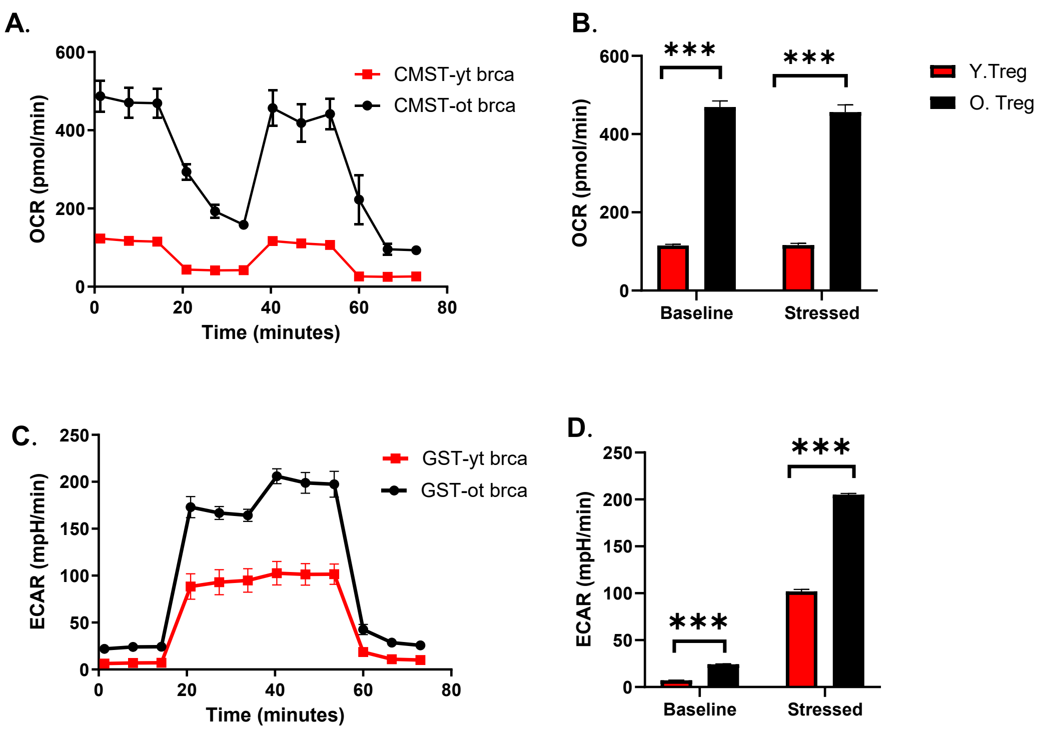

Fig S11: Glycolysis metabolites in Tregs of young and old mice

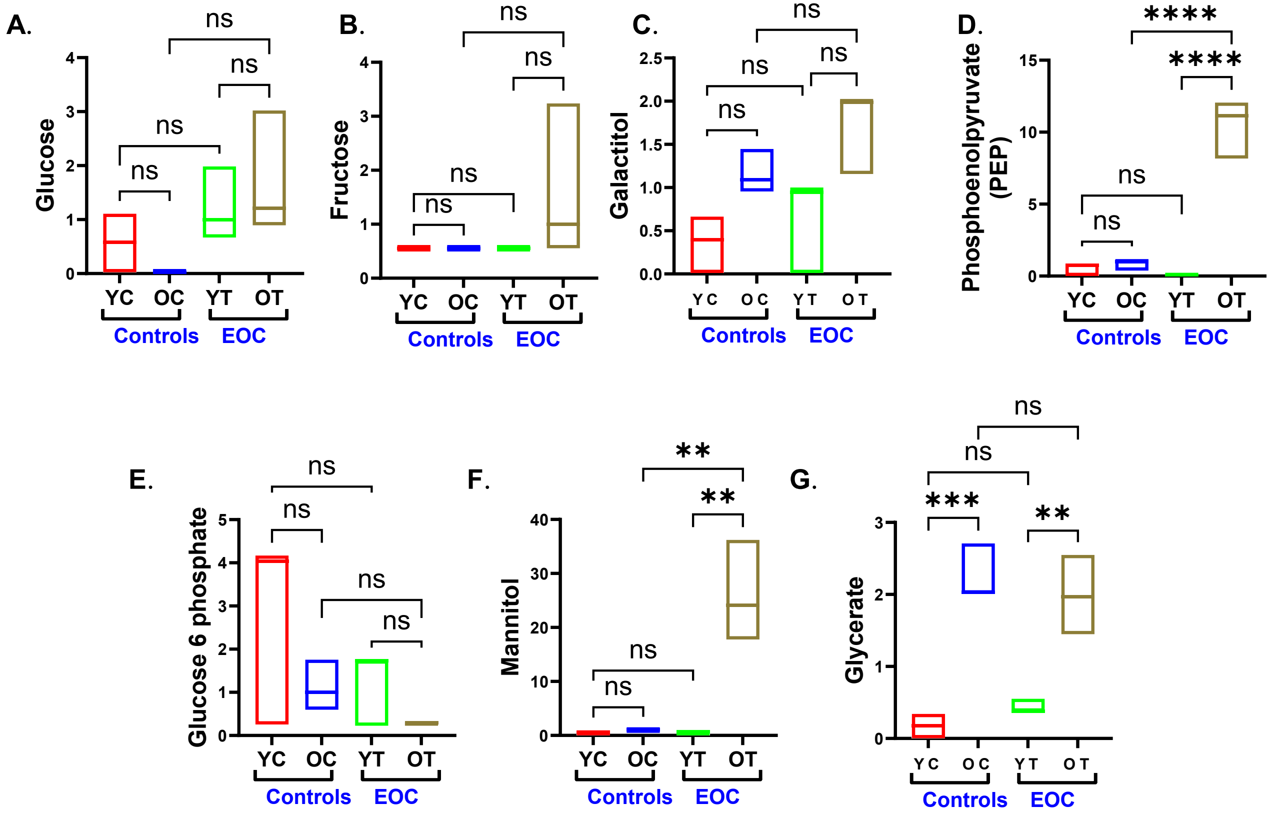

Fig S12: Tumor derived succinate promotes Treg function in aged EOC mice

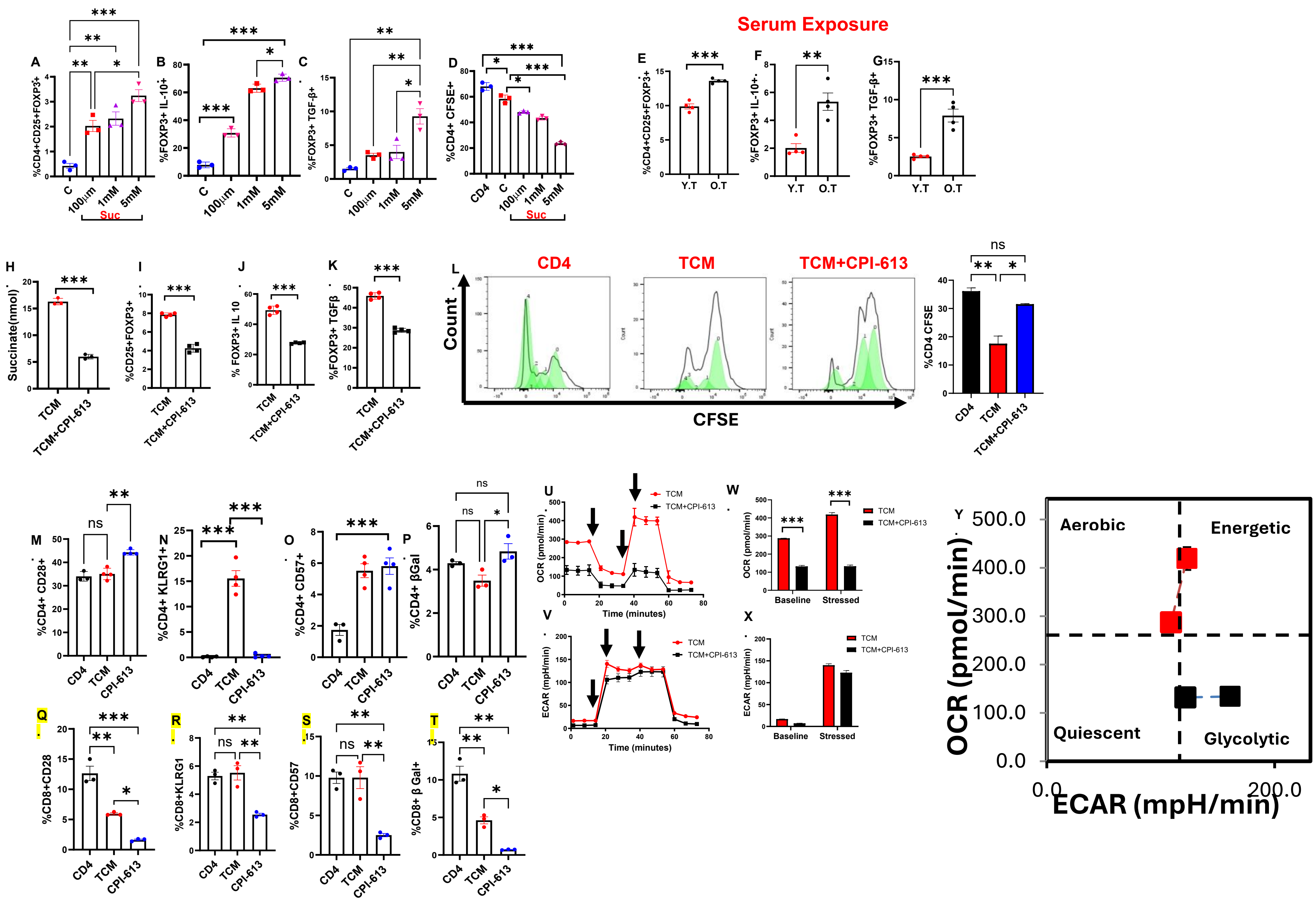

Fig S13: Inhibition of succinate synthesis decreased Treg function

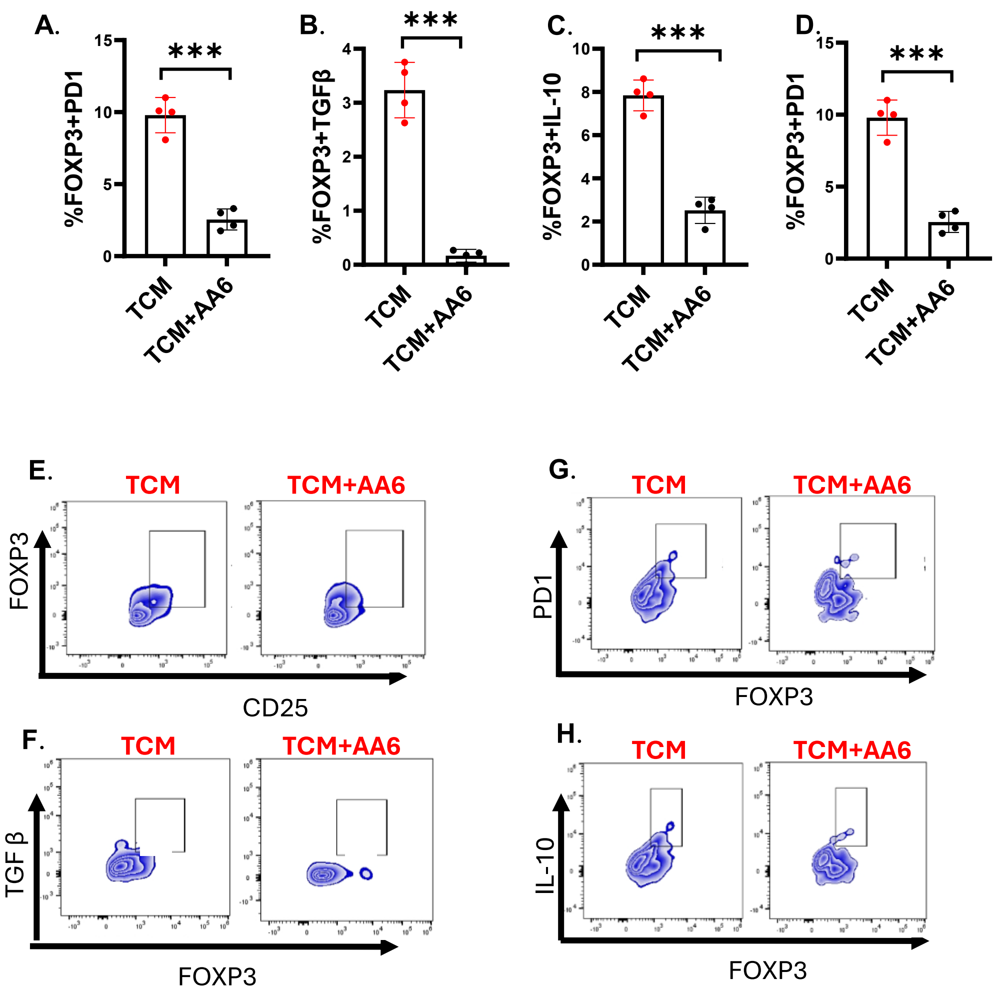

Fig S14: Age-dependent regulatory T cell–mediated suppression differentially impairs CD8^{+} T-cell responses

Old Tregs to young and old CD8

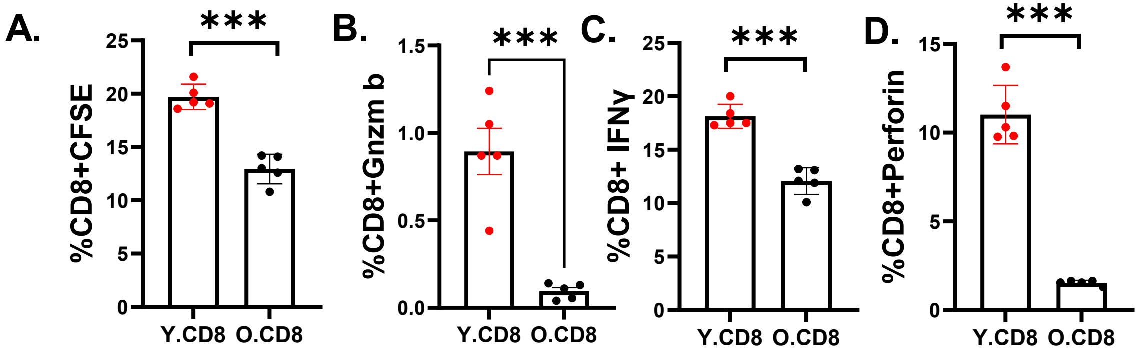

Young Tregs to young and old CD8

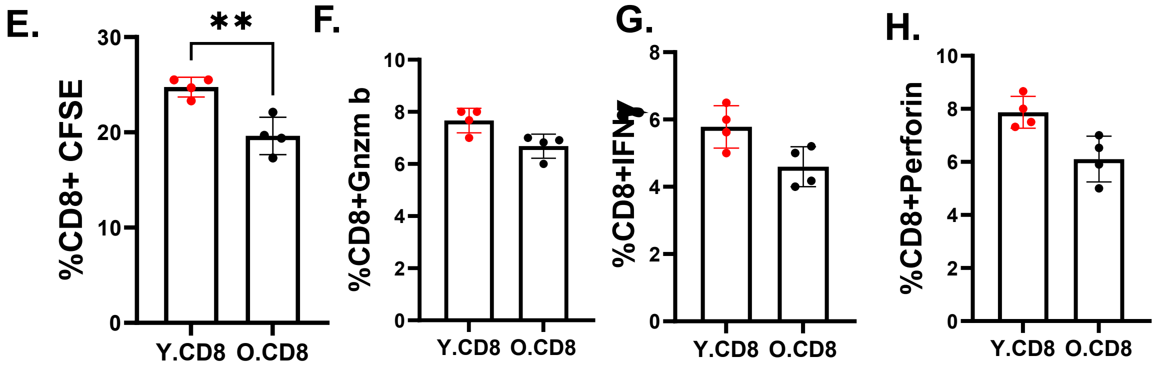

Supplement: Supplementary file 1 — Figure S1: Aging exacerbates preclinical EOC models: ID8‐luc2 cells were injected in young and old EOC mice, (A). Representative BLI images at 4 weeks of tumor inoculation, (B) Bar graph of BLI quantification of images in A. ***p < 0.001, OT compared with YT group by Student's t‐test. Figure S2: Aging enhances tumor promoting metabolic growth factors and Senescence Associated Secretory Phenotype associated inflammation: ID8p53−/− and ID8 P53−/−, BRCA 1−/− EOC cells were injected in YT or OT (n = 10/group), ascites was collected and ELISA was performed for key inflammatory and growth factors. (A–X) Measurement of (A, E) Insulin, (B, F) IGF‐1, (C, G) Adiponectin, (D, H) Leptin, (I, M) IL‐4, (J, N) MCP‐1, (K, O) IL‐10, (L, P) GMCSF, (Q, U) IL‐1β, (R, V) TNFα, (S, W) IL‐6, (T, X) IFN γ, and in ascites collected at week 5 from ID8p53−/− and ID8p53−/−, BRCA 1−/− EOC bearing mice by ELISA (n = 3). *p < 0.05, **p < 0.01, ***p < 0.001, OT compared with YT group by Student's t‐test. Figure S3: Aging induces differential systemic T cell response in response to EOC: ID8p53−/− EOC injected in Y.C, YT or O.C, OT (n = 10/group), blood was collected and processed for immune profiling. (A–I) (A) A representative t‐SNE visualization of markers after gating on single, live, CD45+ CD3+, CD4+, CD8+, IFNγ, Granzyme B (Grz B), perforin and FOXP3 expression. (B) Heatmap represents marker expression of the main T cell subsets in individual samples. Immune profiling was performed in blood of 5 individual mice per group. Bar plots represent the percentage of T cell subsets (C) CD4+, (D) CD4+ IFNγ+, (E) CD4+ CD25+ FOXP3 (F) CD8+, (G) CD8+ IFNγ+, (H) CD8+ Grzb+, (I) CD8+ Perforin+, (J) CD11b+ F4/80, (K) F4/80+ CD38, (L) F4/80+ EGR2, (M) F4/80+ CD206, (N) CD38/EGR2, (O) CD38/CD206, (P) CD38+ iNOS, (Q) EGR2+ Arg1, (R) CD206+ Arg1, (S) CD11b+ GR1. The experiment was repeated twice in two different sets of mouse experiments. *p < 0.05, **p < 0.01, ***p < 0.001. Figure S4: Aging diminishes the T cel [file ACEL-25-e70510-s001.pdf]
